# Facile engineering of silk fibroin capped AuPt bimetallic nanozyme responsive to tumor microenvironmental factors for enhanced nanocatalytic therapy

**DOI:** 10.7150/thno.50486

**Published:** 2021-01-01

**Authors:** Ruihao Yang, Shiyan Fu, Ruidong Li, Lei Zhang, Zhigang Xu, Yang Cao, Hongjuan Cui, Yuejun Kang, Peng Xue

**Affiliations:** 1 State Key Laboratory of Silkworm Genome Biology, School of Materials and Energy, Southwest University, Chongqing 400715, China.; 2 Cancer Center, Medical Research Institute, Southwest University, Chongqing 400716, China.; 3 Chongqing Key Laboratory of Ultrasound Molecular Imaging, Institute of Ultrasound Imaging, Second Affiliated Hospital, Chongqing Medical University, Chongqing, 400010, China.

**Keywords:** Silk fibroin, Nanocatalyst, Tumor theranostics, Biomineralization, Tumor microenvironment

## Abstract

**Background:** Reactive oxygen species (ROS), as a category of highly reactive molecules, are attractive for eliminating tumor cells *in situ*. However, the intrinsic tumor microenvironment (TME) always compromises treatment efficacy. In another aspect, silk fibroin (SF), as a category of natural biomacromolecules, is highly promising for synthesis of metallic nanocrystals via biomineralization.

**Methods:** As a proof-of-concept study, AuPt bimetallic nanozyme derived from bioinspired crystallization of chloroauric acid and chloroplatinic acid was facilely developed in the presence of silk fibroin (SF). Antitumor effects caused by the as-synthesized AuPt@SF (APS) nanozyme were demonstrated in 4T1 tumor cells *in vitro* and xenograft tumor models *in vivo*.

**Results:** APS nanozyme can decompose glucose to constantly supply H_2_O_2_ and deplete intracellular glutathione (GSH). APS nanozyme can simultaneously convert adsorbed O_2_ and endogenic H_2_O_2_ into superoxide radicals (^•^O_2_^-^) and hydroxyl radical (^•^OH), respectively, upon highly efficient catalytic reaction. Subsequently, these cytotoxic ROS cause irreversible damage to the cell membrane, nucleic acid and mitochondria of tumors. Upon fluorescence/photoacoustic (FL/PA)-imaging guidance, remarkable tumor damage based on the current nanoplatform was confirmed *in vivo*.

**Conclusion:** The objective of our investigation is to supply more useful insights on the development of SF-based nanocatalysts, which are specifically responsive to TME for extremely efficient tumor theranostics.

## Introduction

Compared with normal cells, reactive oxygen species (ROS) are significantly upregulated in tumor cells due to increased metabolic rate, mitochondrial malfunction and enhanced activity of the peroxisome 1-4. In another aspect, an excessively high level of ROS might result in irreversible cell destruction under such oxidative stress, via damage to intracellular lipids, proteins and DNA 5-9. Among the representative ROS, the hydroxyl radical (^•^OH), peroxide ion (O_2_^2-^), singlet oxygen (^1^O_2_), superoxide radical (^•^O_2_^-^), etc. have been frequently selected for elimination of tumor cells 10, 11. Recently, emerging nanocatalytic tumor therapy by production of highly cytotoxic ROS based on catalytically active metallic nanoparticles (NPs) has attracted increasing attention in the scientific community 12, 13. In contrast to natural enzymes, these so-called “nanozymes” exhibit many unique advantages, including facile synthesis, reliable catalytic activities under harsh conditions, and ease of separation or purification 14-18. However, the majority of current nanozymes are not capable of realizing a satisfactory therapeutic outcome because they are compromised by unfavorable aspects of the tumor microenvironment (TME), such as upregulated intracellular glutathione (GSH) and local hypoxia 19, 20. Moreover, metallic nanozymes also suffer from relatively poor aqueous stability and undesirable biocompatibility, which remarkably hinder their applications in tumor theranostics 21-23. To this end, it becomes increasingly important to develop facile synthetic strategies for obtaining biocompatible metallic nanozymes, which can effectively circumvent the limitation of TME clues for augmented nanocatalytic therapy.

Silk fibroin (SF), which is extracted from the cocoon of the silkworm (*Bombyx mori*), contains a good number of amino acids with side chains, which supply abundant reactive sites for biological and chemical modifications 24-26. With good biocompatibility, biodegradability and definite metabolic pathways, SF has been extensively used in biomedical and pharmaceutical fields in diversified forms such as films, scaffolds, hydrogels, particles and fibers 27-29. In particular, SF NPs with a large specific surface area exhibit outstanding aqueous stability and can serve as the sacrificial template or reductant for assembly of nanostructures. For instance, crystallization of CaCO_3_ and MnO_2_ can be achieved on the skeleton of SF via biomineralization-inspired metal reduction 30, 31. However, until now, no studies related to the synthesis of novel nanocatalyst through SF-mediated bioinspired crystallization have been conducted. In another aspect, gold (Au) NPs and platinum (Pt) NPs have exhibited remarkable enzymatic activity due to their tiny size and structure-dependent properties and have been applied in bioanalysis, antibiosis and environmental monitoring 32, 33. Specifically, citrate-coated Au NPs can trigger the oxidation of glucose into H_2_O_2_ due to glucose oxidase (GOx)-mimicking activity 34, and Au NPs also display the intrinsic enzymatic activities of catalase and peroxidase 35-37. Similarly, Pt NPs (size < 5 nm) have shown good catalytic activities in mimicking of catalase, peroxidase and uricase 38-40. More importantly, compared with individual Au and Pt NPs, researchers have demonstrated that AuPt bimetallic NPs showed synergistic catalytic activity due to strain and ligand effects, which are closely correlated to modification of the surface electronic structure 41, 42.

**Scheme 1:**
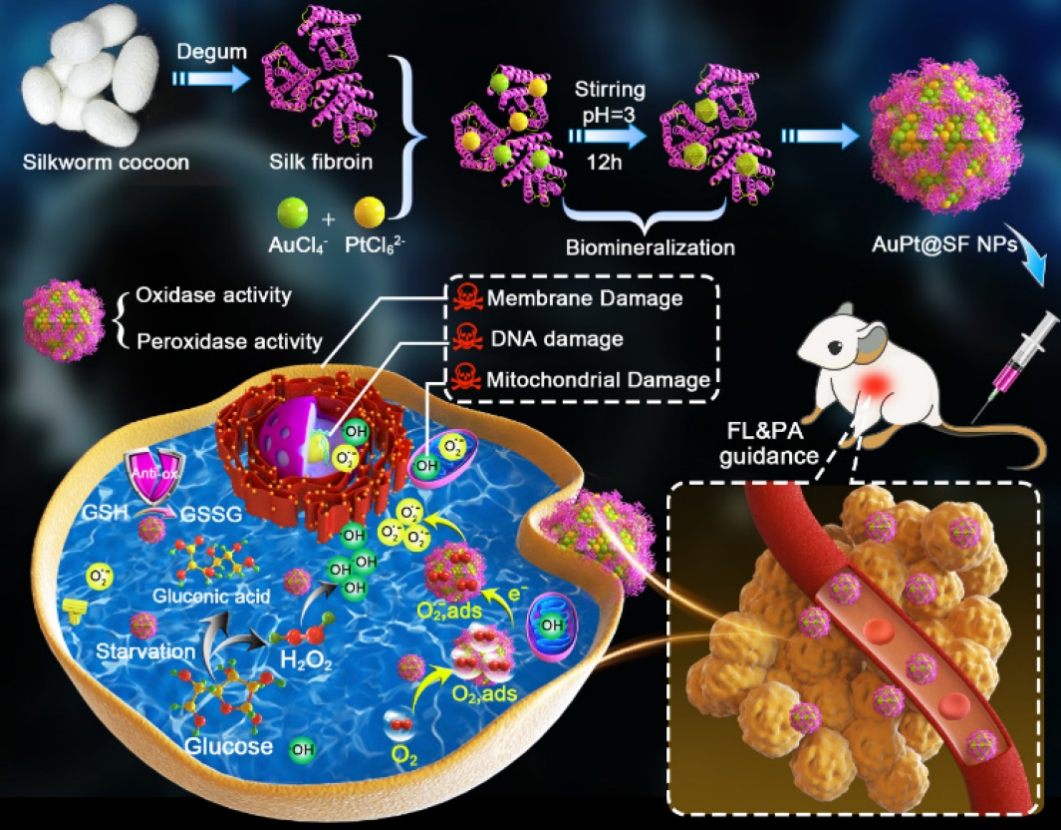
Schematic illustration of the synthesis route for the APS bimetallic nanozyme and its application for enhanced nanocatalytic therapy under fluorescence/photoacoustic (FL/PA) imaging guidance.

Herein, we used SF as the mineralization inducer and sacrificial template to synthesize AuPt@SF (APS) bimetallic nanozyme via one-step reduction, a material that can be used in an enhanced nanocatalytic therapy by overcoming the limitations of TME clues. As shown in Scheme 1, intracellular H_2_O_2_ level can be effectively upgraded through glucose oxidation in the presence of APS. Thereafter, APS nanozyme can convert the adsorbed O_2_ and endogenic H_2_O_2_ into superoxide radicals (^•^O_2_^-^) and hydroxyl radicals (^•^OH), respectively, due to the mimicking activities of oxidase and peroxidase. Additionally, the depletion of reductive glutathione (GSH) into glutathione disulfide (GSSG) further protects the generated ROS to increase the therapeutic efficacy. Such glucose consumption and enhanced ROS production through enzymatic reaction can effectively damage tumor cells through deleterious tumor starvation and irreversible oxidative-stress destruction. Consequently, solid tumors can be eradicated with minimal systemic side effects, benefitting from the decent biocompatibility of capped SF on APS. The observation might offer unique insights for the assembly of novel metallic nanozymes via a SF-inspired crystallization strategy.

## Results and Discussion

**Figure 1:**
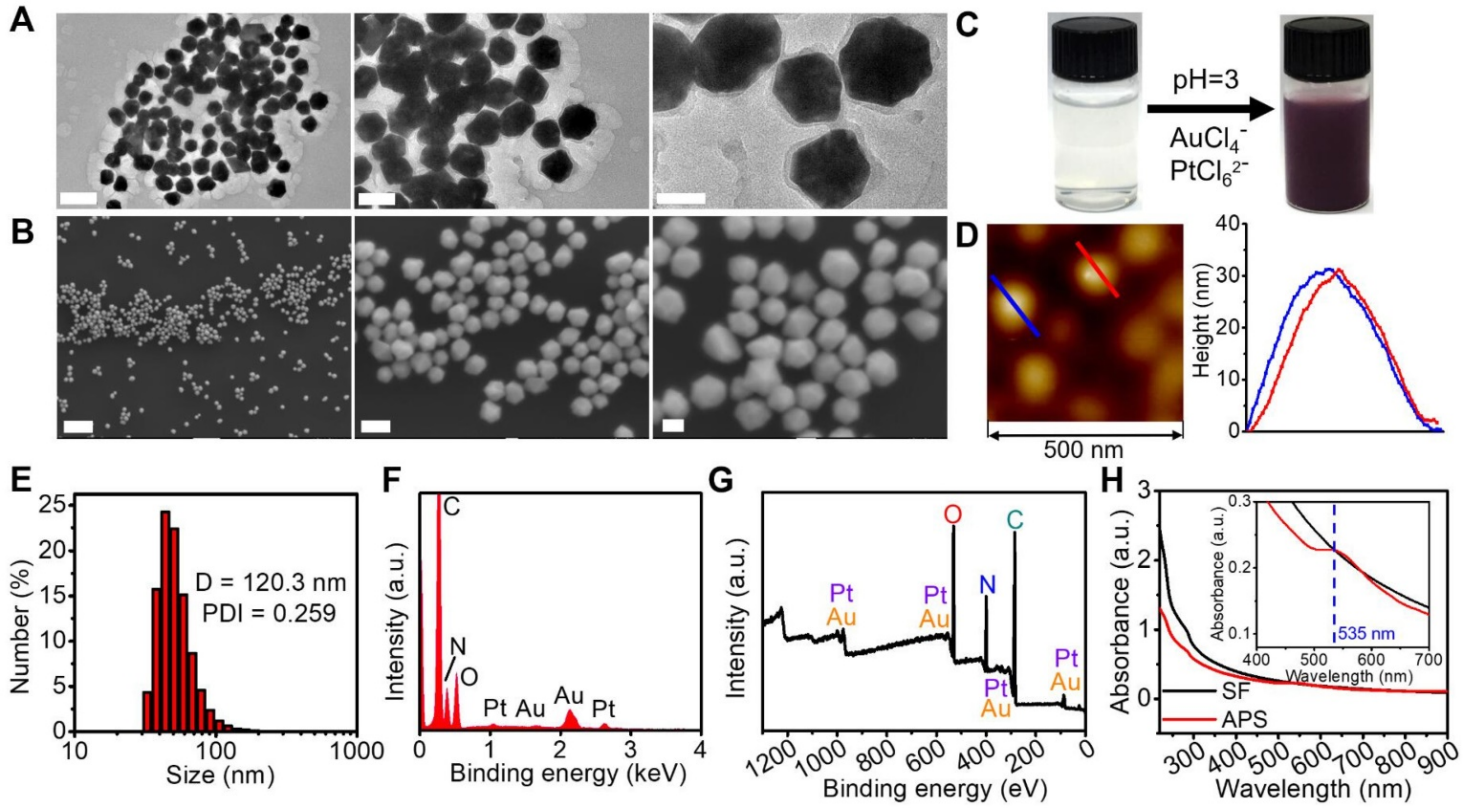
** Structural characterization.** (A) TEM images of APS NPs with multiple magnifications (scale bars: 200, 100 and 50 nm from left to right); (B) SEM images of APS NPs with multiple magnifications (scale bars: 1 µm, 100 and 50 nm from left to right); (C) digital images of SF solution and APS NP dispersion; (D) AFM image of APS NPs; (E) Hydrodynamic diameter of APS NPs measured by dynamic light scattering (DLS); (F) EDS spectrum of APS NPs; (G) XPS full survey spectrum of APS NPs; (H) UV-Vis-NIR absorption spectra of SF solution and APS NPs (inset: local magnified view in the wavelength range of 400-900 nm).

Compared with the morphology of APS NPs obtained from the molar ratio of Au/Pt precursors at 3:1, 2:1, 1:2 and 1:3, a more homogenous and regular profile of APS NPs with uniform distribution was observed at a precursor ratio of 1:1 (Figure 1A and S1). Herein, the precursor ratio of Au/Pt at 1:1 was selected for preparation of APS NPs in this study. The as-prepared APS bimetallic nanoparticles (NPs) exhibited an intriguing nonregular polyhedral structure, as observed from scanning electronic microscopy (SEM) and transmission electronic microscopy (TEM) (Figure 1A and 1B). After bioinspired crystallization in the presence of SF, the homogenous solution of nanozyme displayed a dark purple color (Figure 1C). Based on atomic force microscopy (AFM), the average diameter and height were measured as 68.71 ± 32.8 nm (Figure 1A) and 31.78 ± 1.57 nm (Figure 1D), respectively. In addition, the hydrodynamic size and polydispersity index (PDI) of the nanozyme were 120.3 nm and 0.259, respectively, which is within the valid size range (50-200 nm) for the enhanced permeation and retention (EPR) effect (Figure 1E) 43. Long-term stability of APS NPs under physiological condition was evaluated by monitoring the hydrodynamic diameter in PBS, DMEM and FBS (10%) over seven days, and no dramatic fluctuation of the hydrated size occurred during this period, suggesting excellent stability for circulation in the bloodstream (Figure S2). The energy dispersive spectroscopy (EDS) spectrum exhibited typical peaks of Au and Pt (Figure 1F), which was consistent with the findings in element mapping (Figure S3). Furthermore, elemental composition was quantitatively analyzed using ICP-MS, and the content of Au and Pt was calculated as 27.48% and 21.19% by weight. The X-ray photoelectron spectroscopy (XPS) pattern further demonstrated the successful synthesis of APS nanozyme without any impurities (Figure 1G and S4). To verify the oxygen adsorption property of APS NPs, core level XPS patterns (O1s) of APS NPs and SF were measured for investigation (Figure S5). Obviously, a primary peak centered at 531.2 eV and a satellite peak at 532.7 eV in the APS spectrum can be assigned to oxygen in the combined and adsorbed state, respectively, which concretely demonstrated the existence of oxygen on the APS surface in the form of O_2,ads_ 44, 45. The X-ray diffractometer (XRD) pattern revealed the well-defined lattice plane of Au (JCPDS No.04-0784) due to the formation of the Au/Pt substitution solid solution (Figure S6) 46, 47. The single fiber of SF is composed of four peptides, and amide I and III are detectable using Raman spectroscopy 48. The Raman spectrum of APS NPs displayed scattering bands similar to those of freeze-dried SF in the wavenumber range of 900-1700 cm^-1^, suggesting the existence of SF in the nanozyme (Figure S7). The UV-Vis-NIR spectrum of APS NPs exhibited unique peak bands at 250-300 nm and ~535 nm, which can be assigned to SF protein and Au/Pt atoms, respectively 47, 49 (Figure 1H). The specific surface area and average pore size of APS NPs were measured as 97.254 m^2^/g and 2.6 nm, respectively (Figure S8). Previous reports have revealed probable mechanisms of oxygen adsorption on metallic-based nanostructures. For instance, abundant surface defects might contribute to excessive oxygen adsorption on the transition metal oxide NPs 50. As another example, the high magnitude of surface energy also facilitates the oxygen binding in Au NPs 51 and Pt NPs 52. Considering the highly porous structure and Au-Pt bimetallic composition, both the abundant surface defects and high magnitude of surface energy might have effects on the oxygen adsorption in APS NPs.

**Figure 2:**
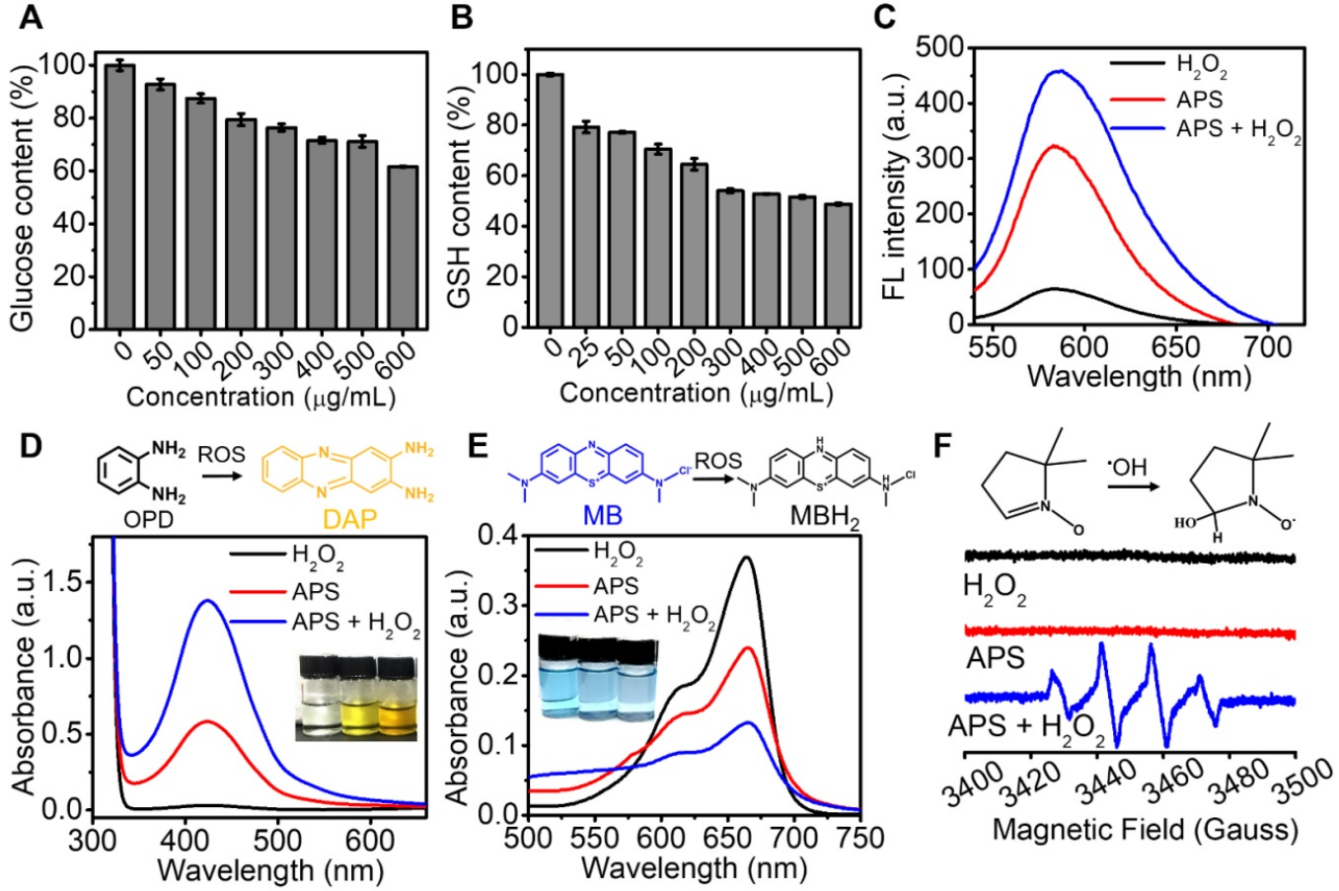
Catalytic activity. (A) Glucose depletion by APS at different concentrations during 8 h incubation; (B) GSH consumption by APS at various concentrations during 4 h incubation; (C) fluorescence spectra of DHE (20 µg/mL), (D) optical absorption spectra of OPD (500 µg/mL) and (E) optical absorption spectra of MB (500 µg/mL) after reacting with diversified agents in PBS (pH = 5.5) for 15 min; (F) ESR spectra of APS NPs upon the addition of DMPO (trapping agent of ^Ÿ^OH).

Considering the decent oxidase-mimicking activity of APS, we further attempted to apply this nanozyme for oxidization of endogenic reductive substances, including glucose and GSH *in vitro*. The glucose level decreased with the increasing concentration of APS, which might potentially achieve tumor starvation and intracellularly create abundant H_2_O_2_ (Figure 2A). As characterized by the DTNB probe, APS also contributed to GSH depletion, which can effectively invalidate the antioxidant system to aggravate cell destruction under oxidative stress (Figure 2B). In the presence of ^Ÿ^O_2_^-^, dihydroethidium (DHE) can be dehydrogenated into ethidium bromide (EB), which can irreversibly bind DNA to produce red fluorescence. A good amount of ^Ÿ^O_2_^-^ was detected after adding APS nanozyme into PBS, as evidenced by the observation of a fluorescence emission increase at 590 nm (Figure 2C). In particular, a higher concentration of ^Ÿ^O_2_^-^ was generated in the presence of H_2_O_2_, which could be ascribed to the upgraded O_2_ level in the reaction system (Figure S9). Furthermore, the ROS generation capability of APS was further demonstrated by measuring the absorption change of o-phenylenediamine (OPD) at 435 nm (Figure 2D). Compared with H_2_O_2_ control, APS alone resulted in an obvious increase of the absorption value, attributed to OPD oxidation by the generated ^Ÿ^O_2_^-^. The most significant ROS generation was observed upon the addition of H_2_O_2_ due to the contribution of ^•^OH created from peroxidation reaction. The peroxidase-mimicking activity of APS was further evaluated by oxidizing the peroxidase chromogenic substrate of methylene blue (MB) (Figure 2E). Relative to the H_2_O_2_ control, the introduction of APS nanozyme resulted in the optical absorption decrease at 670 nm due to oxidation of MB by the produced ROS.

The enzyme-mimicking activity of APS NPs was further evaluated based on Michaelis-Menton steady-state kinetics. The peroxidase-like and GOx-like catalytic activities of APS NPs were compared with the natural enzymes of horseradish peroxidase (HRP) and GOx, respectively. First, the time-course absorbance of APS NPs or HRP (with the equivalent dosage of 40 µg/mL) containing OPD (500 µg/mL) was measured at 435 nm in the presence of H_2_O_2_ at gradient concentrations. Subsequently, the Michaelis-Menten curve was produced based on plotting the relative reaction rate against substrate concentration (Figure S10). Thus, the Michaelis-Menten constant (K_M_) and maximum relative velocity (V_max_) were calculated as 28.148 mM and 6.756 for APS NPs, which were much higher than the values of 15.636 mM and 1.807 for HRP. In another aspect, time-course glucose consumption catalyzed by APS NPs or GOx (with the equivalent dosage of 40 µg/mL) was measured using DNS (λ_max_ = 532 nm) based on spectrophotometry in the presence of glucose at gradient concentrations (Figure S11). Similarly, the K_M_ and absolute V_max_ values were calculated as 45.795 µg/mL and 0.125 µM/s for APS NPs, which were slightly lower than the 60.559 µg/mL and 0.231 µM/s for GOx. These results explicitly indicated that the catalytic activities of APS NPs were comparable to those of the natural enzymes and that these materials might perform even better in harsh environmental conditions due to the intrinsic high stability of the nanozyme 53.

In addition to colorimetric methods, electron spin resonance (ESR) spectroscopy with the spin trap of 5,5-dimethyl-1-pyrroline N-oxide (DMPO) demonstrated the production of short-lived ^•^OH and ^Ÿ^O_2_^-^ mediated by APS in the presence of H_2_O_2,_ as proved by the characteristic signal intensity of 1:2:2:1 in aqueous dispersion and of 1:1:1:1 in methanol dispersion, respectively (Figure 2F and S12). In addition, the H_2_O_2_ level was decreased after incubation with APS nanozyme for 15 min, implying that H_2_O_2_ served as the sacrificial substrate for ROS generation (Figure S13). These results explicitly revealed that APS can effectively facilitate the production of ^Ÿ^O_2_^-^ and ^•^OH under the TME-mimicking condition, and the following mechanisms were suggested, as shown in equation (1) and (2):


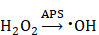
(1)


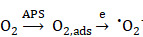
(2)

**Figure 3:**
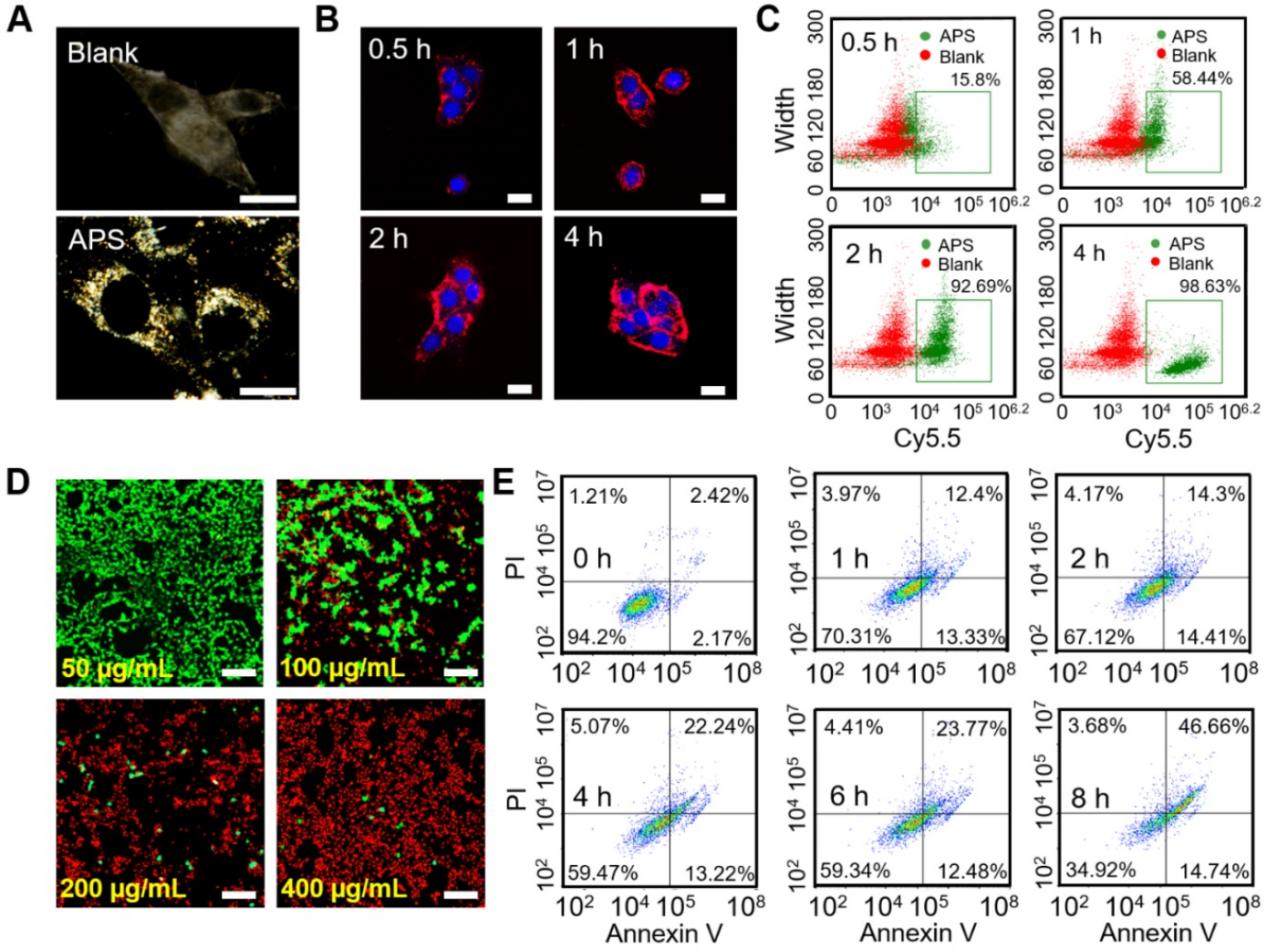
*In vitro* study at cellular level. (A) Dark-field image of 4T1 cells after treatment with APS NPs (400 µg/mL) for 4 h; (B) fluorescence images of 4T1 cells after exposure to Cy5.5-labeled APS NPs (200 µg/mL) for various periods; (C) quantitative cellular uptake of APS NPs characterized by flow cytometry; (D) LIVE/DEAD fluorescence staining of 4T1 cells after incubation with APS NPs for 12 h (scale bar: 100 µm); (E) apoptosis of APS-treated 4T1 cells evaluated by Annexin V/PI staining.

Dark-field imaging is a useful technique for tracing the light-scattering of metallic NPs. As shown in Figure 3A, effective internalization of APS NPs was observed in 4T1 cells after 4 h exposure, as evidenced by the significant signal enhancement in the cytoplasm. In addition, intracellular fluorescence emission was further detected upon coincubation of 4T1 cells and Cy5.5-labeled APS NPs for various periods. Endocytosis of NPs occurred in a time-dependent manner, which was determined by the increased fluorescence intensity of Cy5.5 over time (Figure 3B). Such an NP internalization tendency was also discovered through flow cytometry analysis (Figure 3C and S14). Furthermore, cell uptake was significantly suppressed at a temperature of 4°C or upon the addition of NaN_3_ (inhibitor of ATP hydrolase), implying an energy-dependent process (Figure S15A). Moreover, the clathrin-mediated pathway played a key role in the endocytosis of APS, as proved by the marvelous uptake inhibition caused by chlorpromazine (CPZ) (Figure S15B). Lysotracker Green, a standard lysosome marker, was used to selectively label the lysosome during investigation of the endocytosis of APS NPs. As shown in Figure S16, APS NPs were found to be progressively colocalized with the lysosome during the first 2 h, as evidenced by the gradually increased fluorescence overlap of APS NPs with Lysotracker Green. The acidic environment of the lysosome is highly favorable to reaching an optimum peroxidase- or oxidase-like activity of APS nanozyme. However, colocalization of Cy5.5-labeled APS NPs with the lysosome was decreased after 4 h incubation, as ascribed to lysosomal escape caused by ROS-induced disruption of the lysosomal membrane.

Before evaluating the cytotoxicity of APS NPs, their biocompatibility was first investigated in somatic cells, including L929 murine fibroblasts and human umbilical vein endothelial cells (HUVECs). LIVE/DEAD fluorescence cell staining, 2,7-dichlorodi-hydrofluorescein diacetate (DCFH-DA) assay and JC-1 mitochondrial membrane potential (MMP) assay verified minimal cell death, insignificant ROS generation and negligible damage to mitochondria, respectively, even at an extremely high APS concentration of 400 µg/mL (Figure S17-S19). The MTT assay quantitatively demonstrated such extremely low toxicity toward normal cells, further suggesting the admirable biosafety of APS (Figure S20). However, the cytotoxicity caused by APS NPs toward 4T1 cells was further investigated *in vitro*. Compared with somatic cells, significant tumor cell damage was induced by APS at concentrations over 200 µg/mL (Figure S21). Additionally, LIVE/DEAD fluorescence cell staining further manifested dose-dependent cytotoxicity toward 4T1 cells, as indicated by the progressively enhanced red fluorescence at an APS concentration of up to 400 µg/mL (Figure 3D). Moreover, APS-mediated tumor cell apoptosis was aggravated with prolonged incubation time of up to 8 h (Figure 3E).

Inspired by the ROS generation capacity of APS NPs, we speculated that the high amount ROS could serve as the essential factor that led to the destruction of tumor cells. Therefore, the production of ROS in 4T1 cells was first evaluated through DCFH-DA assay (Figure S22). Vivid green fluorescence was observed in APS-treated tumor cells after 4 h administration, suggesting efficient intracellular ROS generation. In particular, a good amount of internal ^•^O_2_^-^ was detected via the DHE fluorescence probe (Figure S23). To understand the underlying mechanisms of the ROS-activated cell killing effect, the status of ROS-sensitive subcellular components, including the cell membrane, DNA and mitochondria, were evaluated after APS administration. Specifically, methane dicarboxylic aldehyde (MDA), recognized as the products of membrane lipid peroxidation, was almost 40-fold greater than that of blank control group at 8 h, implying serious cell membrane damage (Figure S24). In addition, the Comet assay revealed remarkable APS-induced DNA fragmentation, as evidenced by the markedly long comet tails after treatment (Figure S25). Furthermore, strong green fluorescence representing the JC-1 monomer was detected in 4T1 cells, indicating prominent mitochondria dysfunction caused by APS NPs (Figure S26). Due to the peroxidase activity of APS, the intracellular GSH level was downgraded, as measured by Ellman's reagent, which can dramatically invalidate the self-defense of tumor cells under oxidative stress (Figure S27A). In addition, the intracellular ATP level was considerably reduced after 4 h incubation with APS (Figure S27B). ATP deprivation might lead to apoptosis or necrosis of tumor cells, which is promoted by mitochondrial impairment 54, 55.

**Figure 4:**
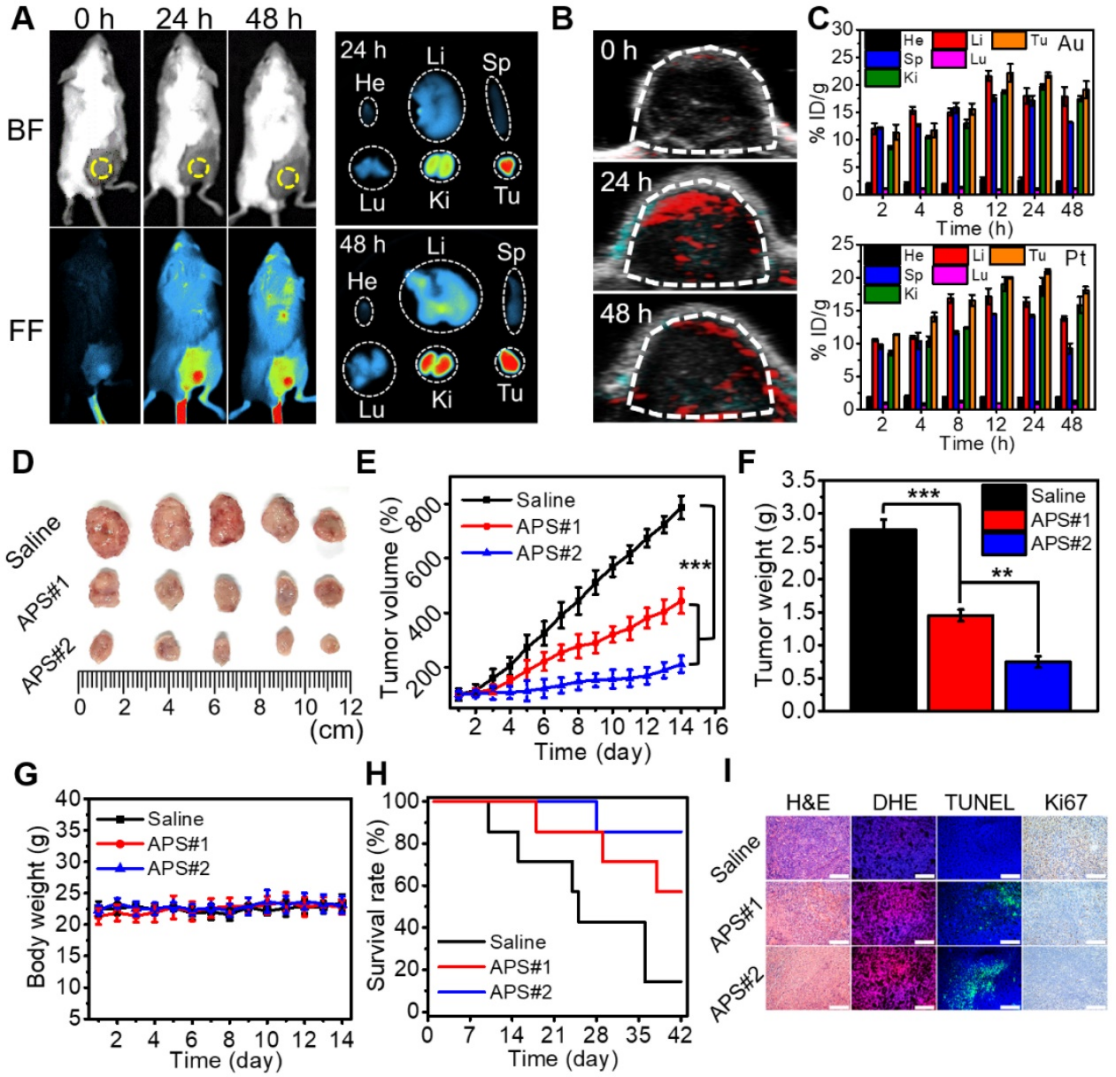
Study on animal models. (A) Fluorescence images of tumor-bearing mice at 24 and 48 h after intravenous injection of Cy7.5-labeled APS NPs and *ex vivo* images of excised tumor and major organs (He: heart, Li: liver, Sp: spleen, Lu: lung, Ki: kidney, Tu: tumor); (B) PA images of tumor region at 24 and 48 h after APS administration; (C) biodistribution of Au and Pt content after APS treatment; (D) photograph of excised solid tumor on day 14; (E) variation in tumor volume within 14 days (^***^p<0.001); (F) average weight of dissected tumors on day 14 (^**^p<0.01, ^***^p<0.001); (G) change in mouse body weight; (H) mouse survival rate; (I) histological analysis of tumor section collected on day 14 (scale bar: 200 µm).

Versatile imaging navigation not only contributes to the understanding of agent biodistribution but also favors accurate localization of cancerous tissue. The maximum enrichment of Cy7.5-labeled APS NPs within the tumor region was observed at 24 h postadministration, as evidenced by the strongest local fluorescence emission (Figure 4A and S28). By taking advantage of the photothermal response, the PA signal intensity of APS NPs increased with increasing concentration ranging from 15.6 to 500 µg/mL (Figure S29 and S30). Furthermore, PA images of tumor site were collected at predefined time points (24 and 48 h) postinjection, and the local PA signal intensity reached a peak level at 24 h (Figure 4B and S31). The biodistribution of APS NPs was further assessed by quantifying the Au and Pt content in major organs and tumors using inductively coupled plasma atomic emission spectroscopy (ICP-AES), and progressive accumulation was discovered in the tumor region over 24 h, as ascribed to the EPR effect (Figure 4C). The content of APS NPs in the tumor tissue was higher overall than that in other organs, which implied an excellent tumor targeting behavior. Apart from EPR effect, several other factors might also contribute to tumor-specific enrichment of APS NPs. A previous study revealed that SF coating might enhance adhesive targeting based on the interaction between SF and the pericellular matrix, where particular receptor-ligand conjugation is not imperative 56. Moreover, the β-barrels of SF can act as porins to attach, penetrate and destabilize the cell membrane, thus creating an access port for intracellular localization of APS NPs 57. In these cases, a high rate of interstitial space permeation of APS NPs could be significantly attenuated in the tumor tissue, which in turn increased local accumulation and retention. The hemolysis rate caused by APS NPs (6 h incubation) was less than 5% even at concentrations as high as 800 µg/mL, implying excellent hemocompatibility (Figure S32). Moreover, all of the primary indicators of the blood routine test were located in the normal reference range, indicating that APS NPs triggered negligible systemic toxicity after administration (Figure S33). These results demonstrated a decent biosafety of APS for potential clinical applications.

To evaluate the antitumor effect of APS NPs *in vivo*, saline (100 µL), APS#1 (100 µL, 1 mg/mL) and APS#2 (100 µL, 2 mg/mL) were intravenously injected into BALB/c mice bearing 4T1 tumors. Tumor growth was obviously suppressed in the APS-treated groups over 14 days, and APS#2 administration achieved improved treatment efficacy compared with APS#1 treatment, implying a dose-dependent therapeutic effect (Figure 4D-F). The mouse body weight was not significantly altered during the treatment of all groups (Figure 4G). The survival rates were remarkably increased in the APS-treated groups, in comparison with saline group (Figure 4H). Hematoxylin and eosin (H&E) staining verified the prominent necrosis in the APS-treated groups, including nuclear condensation and cell shrinkage (Figure 4I). DHE staining displayed a high ROS level in tumorous tissue mediated by APS. Moreover, APS treatments also resulted in the increase in apoptotic cells and inhibition of cell proliferation, as proved by TUNEL and Ki67 staining, respectively. In contrast, no observable damage appeared in normal tissue due to the highly tumor-specific catalytic activity of APS NPs (Figure S34). The systemic circulation half-life of APS NPs was less than 6 h, and a possible excretion pathway of these foreign substances was suggested through the reticuloendothelial systems (Figure S35). It can be inferred that the long-term stability and circulation half-life of APS NPs are expected be dramatically increased under physiological conditions, in contrast to tiny-sized Au and Pt nanosatellites 58. In another aspect, silk fibroin (SF) might enhance the reprogramming of tumor associated macrophages toward the M1 type, manifesting the potential of APS NPs acting as an immunologic adjuvant for tumor regression 59.

## Conclusion

In summary, we successfully developed the APS nanocatalyst through one-step SF-inspired oriental crystallization. By virtue of unique TME factors, this new agent can realize tumor-specific ROS generation for highly efficient nanocatalytic therapy. APS NPs are capable of increasing intracellular H_2_O_2_ through glucose decomposition for upgrading of the ROS level and depriving local GSH to resist ROS scavenging. The selective production of ROS led to tumor suppression both *in vitro* and *in vivo*, with reliable biosafety. FL/PA bimodal imaging facilitated optimization of the treatment recipes. The objective of our investigation is to supply more useful insights on the development of SF-based nanocatalysts in tumor theranostics for future endeavors by taking advantage of both the unique properties of SF and biomimetic enzymatic activity of the nanocatalyst.

## Supplementary Material

Supplementary figures and tables.

## Abbreviations

APS: AuPt@SF
AFM: atomic force microscopy
ATP: adenosine triphosphate
CPZ: chlorpromazine
DHE: dihydroethidium
DLS: dynamic light scattering
DMPO: 5,5-dimethyl-1-pyrroline N-oxide
DCFH-DA: 2,7-dichlorodi-hydrofluorescein diacetate
EB: ethidium bromide
ESR: electron spin resonance
EPR: enhanced permeation and retention
EDS: energy dispersive spectroscopy
FL/PA: fluorescence/photoacoustic
GSH: glutathione
GSSG: glutathione disulphide
GOx: glucose oxidase
HUVECs: human umbilical vein endothelial cells
HRP: horseradish peroxidase
ICP-AES: inductively coupled plasma atomic emission spectroscopy
MB: methylene blue
MMP: mitochondrial membrane potential
MDA: methane dicarboxylic aldehyde
NP: nanoparticles
OPD: o-phenylenediamine
ROS: reactive oxygen species
SF: silk fibroin
SEM: scanning electronic microscopy
TEM: transmission electronic microscopy
TME: tumor microenvironment
XPS: X-ray photoelectron spectroscopy
XRD: X-ray diffractometer
