# Supplementary material for: Facile engineering of silk fibroin capped AuPt bimetallic nanozyme responsive to tumor microenvironmental factors for enhanced nanocatalytic therapy
Source: Theranostics. 2021 Jan 1;11(1):107–16. doi: 10.7150/thno.50486 (PMC7681078; doi:10.7150/thno.50486)
Supplement: Supplementary file 1 — Supplementary figures and tables. [file thnov11p0107s1.pdf]

Supplementary Material

Facile Engineering of Silk Fibroin Capped AuPt Bimetallic Nanozyme Responsive to Tumor Microenvironmental Factors for Enhanced Nanocatalytic Therapy

Ruihao Yang,[1] Shiyan Fu,[1] Ruidong Li,[1] Lei Zhang,[2] Zhigang Xu,[1] Yang Cao,[3] Hongjuan Cui,[2] Yuejun Kang,[1], Peng Xue[1, *]

[1] State Key Laboratory of Silkworm Genome Biology, School of Materials and Energy, Southwest University, Chongqing 400715, China.

[2] Cancer center, Medical Research Institute, Southwest University, Chongqing 400716, China.

[3] Chongqing Key Laboratory of Ultrasound Molecular Imaging, Institute of Ultrasound Imaging, Second Affiliated Hospital, Chongqing Medical University, Chongqing, 400010, China.

Corresponding author:

*Email: xuepeng@swu.edu.cn (P. Xue)

Experimental Section

Chemicals

Bombyx mori cocoons were provided by Institute of Sericulture and Systems Biology, Southwest University (China). Chloroauric acid trihydrate, chloroplatinic acid, calcein AM, propidium iodide (PI), methylene blue (MB), 3,5-dinitrosalicylic acid (DNS), and formalin solution (neutral buffered, 10%) were obtained from Sigma-Aldrich (USA). Hydrogen peroxide (H_2O_2, 30%), calcium chloride dehydrate ($CaCl_2 \cdot 2H_2O$, >99%), hydrochloric acid (HCl, 37%), sodium hydroxide (NaOH, >97.0%), thiazolyl blue tetrazolium bromide (MTT, 98%), glutathione (GSH), dihydroethldum (DHE), trichloroacetic acid (TCA), thiobarbituric acid (TBA), chlorpromazine (CPZ), nystatin, amiloride, methyl-beta-cyclodextrin (Me-β-CD) and dimethyl sulfoxide (DMSO) were acquired from Shanghai Aladdin Bio-Chem Technology Co., Ltd. (China). H_2O_2 quantitative assay kit was purchased from Sangon Biotech (Shanghai) Co., Ltd (China). Dulbecco's modified eagle's medium (DMEM), TrypLE^{TM} Express enzyme, 4',6-diamidino-2-phenylindole (DAPI), fetal bovine serum (FBS), penicillin (10000 U/mL)/streptomycin (10000 μg/mL), LIVE/DEAD® viability/cytotoxicity kit, singlet oxygen sensor green (SOSG), 5,5'-dithiobis (2-nitrobenzoic acid) (DTNB) and phosphate buffered saline (PBS) were supplied by Thermo Fisher Scientific, Inc. (USA). 2', 7'-dichlorofluorescein diacetate (DCFH-DA) was obtained from GEN-VIEW Scientific Inc. (USA). TUNEL apoptosis assay kit, ATP assay kit, Ki67 cell proliferation kit and proteinase K were purchased from Beyotime Biotechnology (China). DNA damage assay kit was obtained from Nanjing Jiancheng Bioengineering institute. Kunming (KM) mice and BALB/c mice models were purchased from Chongqing ENSIWEIER Laboratory Animal Co., Ltd (China). Deionized (DI) water (18.2 MΩ·cm) was purified by a Milli-Q Synthesis A10 purification system (Molsheim, France).

Preparation of silk fibroin (SF) solution from *Bombyx mori* cocoons

SF solution was prepared according to a typical method as previously described.[1-3] Briefly, *Bombyx mori* cocoons were sliced into thin pieces with the size of 1~2 cm^2. Then, these cocoon slices were submerged into 0.5% Na_2CO_3 solution under the boiling state for

30 min to eliminate the gum-like silk sericin. The cotton like precipitation were thoroughly washed with DI water and incubated in a drying oven at 37°C overnight. Then, 1 g silk fiber was mixed with in 125 g Ajisawa's reagent containing $CaCl_2$, ethanol, and DI water (molar ratio at 1:2:8) for 2 h at 90°C. Impurities such as small molecules and salts were removed from the SF solution on the basis of traditional dialysis technique. Finally, the regenerated SF solution was stored at 4 °C prior to use.

Preparation of APS NPs

The synthesis of APS NPs was inspired according to previously reported biomineralization techniques.[4,5] $HAuCl_4$ (3 mL, 6 mM) and H_2PtCl_6 (3 mL , 6 mM) were mixed to form a homogenous solution, which was further introduced into SF solution (6 mL, 4 mg/mL) under stirring at room temperature. Afterwards, pH value of the reaction system was adjusted to three by dropwise adding HNO_3 solution (1.5 wt%). During another incubation for 24 h, the color of the mixture gradually changed from yellow to purple. At last, as-synthesized APS NPs were purified through traditional dialysis technique by using Slide-a-Lyzer dialysis cassette (MWCO 3500, Pierce) against 1000 mL of water for 12 h.

Structural characterizations

Transmission electron microscopy (TEM, JEM-2100, JEOL, Japan) and field emission scanning electron microscopy (FESEM, JSM-7800F, JEOL, Japan) were performed to observe the sample morphology. The particle height was determined through an atomic force microscope (AFM, Dimension ICON-BRUKER, Germany), and hydrodynamic size was monitored via dynamic light scattering (DLS) using a ZetaSizer (Nano ZS90, Malvern Instruments, UK). UV-vis-NIR absorption spectra were recorded through a spectrophotometer (UV-1800, Shimadzu, Japan). Raman spectra were measured by using LabRAM HR Evolution (HORIBA, Japan). Crystallographic form was analyzed through an X-ray diffractometer (XRD-7000, Shimadzu, Japan) with CuK_α radiation (λ=1.5406 Å), and surface chemistry was determined by X-ray photoelectron spectroscopy (XPS) using an X-ray photoelectron spectrometer (ESCALAB 250Xi, Thermo Fisher Scientific, USA). Brunauer–Emmett–Teller (BET) surface area and pore volume were quantified by recording the nitrogen adsorption–desorption isotherm through a Quantachrome Nova

1200e analyzer. The content of Au and Pt in APS NPs was determined by inductively coupled plasma mass spectrometry (ICP-MS; XSeriesII, Thermo Scientific).

Enzymatic activity of APS NPs

Glucose depletion activity: Glucose consumption was conducted by using DNS reagent. Briefly, $KNaC_4O_6 \cdot 4H_2O$ (36.4 g) was dissolved in 100 mL DI water, followed by introducing $C_7H_4N_2O_7$ (1.26 g), NaOH (4.25 g) and phenol (1 g) into the above solution. Working solution of DNS was prepared by adding 200 mL DI water into 100 mL previous stock solution, and stored in dark. To perform the testing, various concentrations of APS dispersion (1 mL, 0~500 µg/mL) was added into 1 mL glucose solution (10 µg/mL), and reaction was allowed to proceed for 8 h at 37°C. Thereafter, as-collected supernatant (1 mL) was diluted by two folds, followed by the addition of DNS working solution (3 mL). At last, optical absorption intensity at 532 nm was measured using a microplate reader (Infinite M200 PRO, TECAN, Switzerland).

GSH deprivation activity: GSH consumption was measured by standard Ellman reagent. Briefly, Ellman working reagent was prepared by dissolving DTNB (40 mg) into 10 mL PBS (0.1 M, pH = 8.0) containing 1 mM EDTA. To perform the detection, 1 mL APS solution (0~600 µg/mL) was mixed with 1 mL GSH (0.2 mg/mL), and the reaction system was under gent shaking at 37°C for 4 h. Next, 400 µL Ellman reagent was added into the above mixture, and another incubation was continued for 30 min. After that, optical absorption intensity of supernatant was measured at 412 nm via a microplate reader. To evaluate intracellular GSH consumption, 4T1 tumor cells (1×10^4 per well) in a 96-well plate were exposed to APS NPs at different concentrations for 4 h. Then, the cells were treated by TCA solution at 4°C for 30 min. Finally, intracellular GSH deprivation was spectrophotometrically determined based on abovementioned Ellman reagent reaction.

ROS generation capacity: 1 mL APS NPs (40 µg/mL) were dispersed in 5 mL PBS (pH 5.5, 0.1M), containing MB (500 µg/mL), OPD (500 µg/mL) or DHE/DNA (20 µg/mL^{-1}: 5 µg/mL^{-1}) mixture. H_2O_2 concentration was set as 10 mM for comparison purpose. After the reaction for 15 min, variations of optical adsorption at 435 nm (OPD) and 670 nm (MB)

were recorded and analyzed by using a spectrophotometer (UV-1800, Shimadzu, Japan). Fluorescence spectra (λ_{ex}: 520 nm, λ_{em}: 610 nm) for DHE testing were monitored by using a microplate reader. To determine the generation of $^{\bullet}OH$, electron spin resonance (ESR) spectrum was measured by using a Bruker EMX Nano spectrometer, upon the addition of DMPO (10 mg/mL) as the trapping agent. In another aspect, H_2O_2 consumption by APS NPs was measured by a H_2O_2 quantitative assay kit (water-compatible). Briefly, 1 mL APS (40 µg/mL) was incubated with 10 mL H_2O_2 (10 mM) for 15 min, followed by the measurement of H_2O_2 level change following the manufacturer's protocol.

Cellular uptake and internalization

To analyze the endocytosis of APS NPs, Cy5.5 as a fluorescence dye was covalently labeled based on a typical strategy at first. [6] Briefly, 5 mL Cy5.5-NHS (5 mg/mL) was mixed with 10 mL APS (400 µg/mL), and the mixture was under stirring for 24 h in dark. Cy5.5-labeled APS was thereby obtained through traditional dialysis technique. To study cellular uptake, 4T1 cells were incubated in a 24-well plate at an initial seeding density of 7.5×10^4 per well. Afterwards, the previous medium was replaced by 500 µL of fresh medium containing Cy5.5-labeled APS NPs (200 µg/mL). After 0.5, 1, 2 or 4 h, the cells were rinsed with PBS and observed under laser confocal scanning microscope (LSCM, LSM800, Zeiss). In another aspect, intracellular fluorescence emission was further detected and analyzed by using flow cytometry (NovoCyte TM 2060R, ACEA Biosciences, USA). To figure out the underlying mechanism of APS internalization, 4T1 cells were exposed to NaN_3 (0.1%, w/v), Nystatin (50 µg/mL), amiloride (2 mM), CPZ (20 µg/mL) and Me-β-CD (0.5 mM), followed by the incubation with APS NPs (400 µg/mL) for 4 h. At last, cell uptake efficiency was calculated based on flow cytometry.

To perform dark-field imaging, 0.5 mL 4T1 cells (1×10^4 per mL) were seeded on a micro slide. After incubation overnight, the cells were treated by 0.5 mL APS NPs (200 µg/mL) for 4 h, followed by gently rinsing to remove loosely bound particles. The cells were fixed by formalin solution (neutral buffered 10%) for 2 h, and examined under a BX51 optical microscope (Olympus, Japan).

Biosafety of APS NPs

MTT assay: Biocompatibility of APS NPs was firstly evaluated by using humanized umbilical vein endothelial cells (HUVECs) and murine L929 fibroblasts (L929 cells). Briefly, cells were seeded in a 96-well culture plate at an initial seeding density of 1×10^4 per well. After 12 h incubation, cells were exposed to the medium containing APS NPs at various concentrations (25~700 µg/mL). Subsequent to another incubation for 24 h, 200 µL MTT solution (250 µg/mL) was introduced into each well to replace the old medium. After 6 h reaction, the supernatant was discarded, followed by the addition of 150 µL DMSO into each well. Then, optical absorption intensity was measured at 490 nm and 630 nm using a microplate reader (SPARK 10M). Cell viability was calculated based on the following formula:

$$\text{Cell viability (\%)} = \frac{\mathrm{OD}_{490nm}\,sample - \mathrm{OD}_{630nm}\,sample}{\mathrm{OD}_{490nm}\,blank - \mathrm{OD}_{630nm}\,blank} \times 100 \quad (1)$$

Hemolytic test: Whole blood sample was collected from the orbital venous plexus of KM mice. After the concentration at 3000 rpm for 5 min (4°C), erythrocytes were harvested at the bottom of centrifuge tubes. Then, the erythrocytes were rinsed with 1×PBS for four times, and re-suspended in 0.5 mL of 1×PBS. Afterwards, 0.5 mL of APS at various concentrations (50, 100, 200, 400 and 800 µg/mL) was added into the tube containing erythrocytes, followed by an incubation for 0.5, 2 and 6 h. After the centrifugation at 8000 rpm for 5 min, the optical intensity of supernatant was measured at 570 nm using a UV-vis spectrophotometer (UV-1800, Shimadzu, Japan).

Blood routine test: KM mice (4~6 weeks, 25 g) were intravenously injected with saline buffer or APS NP dispersion (100 µL, 10 mg/mL). Next, the blood specimen was collected from the orbital venous plexus at day 1, 3, 7, and 14. Key blood index were measured by using an automatic hematology analyzer (NC-2600Vet, Mindary, China).

Cytotoxicity study *in vitro*

Cytotoxicity of APS towards 4T1 cells was firstly evaluated by using MTT assay. 4T1 cells in a 96-well plate (1×10^4 per well) were incubated overnight, and then exposed to APS NPs (25~700 µg/mL) for 24 h. Cell viability was measured on the basis of standard MTT assay

as abovementioned. In another aspect, LIVE/DEAD cell staining was further conducted to directly visualize cell survival status. Briefly, 4T1 cells in a 12-well plate (1×10^5 per well) were incubated overnight, and treated by APS NPs (0~400 μg/mL) for 12 h. After co-stained by calcein AM and PI (following the manufacturer's protocol), the cells were examined through LSCM. To analyze cell apoptotic status after administration, 4T1 cells in a 12-well plate (1×10^5 per well) were cultured overnight, and exposed to APS NPs (400 μg/mL) for 1, 2, 4, 6 and 8 h. Then, the cells were digested by trypsin and re-suspended in PBS containing Ca^{2+} and Mg^{2+}. After being stained by annexin V-FITC and PI, intracellular fluorescence emission was analyzed by using flow cytometry.

Intracellular ROS generation

To monitor the intracellular ROS generation after APS treatment, 4T1 cells in a 24-well plate (1×10^5 per well) were firstly cultured overnight, and treated by APS NPs for various periods. Afterwards, the cells were stained by DCFH-DA (10 μM, 0.5 h) or DHE (20 μM, 2 h), and analyzed by LSCM.

Cell damage at subcellular level

Comet assay: Comet assay was exploited to evaluate the DNA damage of tumor cells based on measuring the tail length of fluorescence comet.[7] Briefly, 100 μL normal melting point agarose (NMA) in the liquid form (45°C) was added on a frozen glass slide. After solidification at 4°C, low melting point agarose (LMA) containing APS-treated 4T1 cells (1×10^4) were uniformed deposited onto the NMA surface. After solidification, as-prepared glass slides were submerged into lysis buffer (90% DMSO) at 4°C for 2 h, and then placed into a horizontal electrophoresis tank containing DNA unwinding buffer (1 mM EDTA, 300 mM NaOH) for 30 min. After embedding, electrophoresis was conducted at 25 V for 30 min. Subsequently, the glass slides were neutralized by Tris-HCl buffer (pH = 7.5), and stained by PI (50 μM), followed by the examination under LSCM.

MDA test: Cell membrane damage was evaluated through MDA test.[8] Briefly, 4T1 cells in a 6-well plate (3×10^5 per well) were cultured at 37°C overnight. After undergoing various treatments, the cells were digested and incubated with TBA in boiling water for 10 min.

After centrifugation at 4000 rpm for 15 min, optical absorption intensity of supernatant (450, 532 and 660 nm) was analyzed by using a microplate reader (SPARK 10M). And, MDA content was quantified based on a recommended formula (2).

$$\mathrm{MDA}\ (\mathrm{mmol/g}) = 6.452 \times (A_{532} - A_{660}) - 0.559 \times A_{450} \quad (2)$$

JC-1 assay: Mitochondrial damage was evaluated by using fluorescence dye JC-1.[9] After the treatment of APS NPs for various periods, 4T1 cells were stained by JC-1 fluorescence probe for 25 min, and intracellular fluorescence emission was observed through FITC and Cy3 channels of LSCM.

Tumor model establishment

All the animal experiments were carried out under the supervision of the Institutional Animal Care and Use Committee (IACUC) of Southwest University and complied with the National Guide for Care and Use of Laboratory Animals. 4T1 cells (1×10^6 in saline) were subcutaneously inoculated on the dorsal side of a female BALB/c mouse (6 weeks, ~25 g), which was continuously bred till tumor volume reached ~250 mm^3. The tumor volume was dynamically monitored and calculated based on equation (3).

$$V = a \times b^2 \times 0.5 \quad (3)$$

Where "a" represents the longest dimension and "b" donates the shortest dimension of the tumor.

Biodistribution and pharmacokinetics *in vivo*

To understand the biodistribution of APS NPs *in vivo*, Cy7.5-NHS was first labeled through covalent conjugation. Briefly, Cy7.5-NHS (5 mL, 5 mg/mL) was mixed with APS NPs (10 mL, 400 μg/mL), and the reaction was continued for 24 h under stirring. Then, Cy7.5-labeled APS NPs (100 μL, 1 mg/mL in saline) were intravenously injected into BALB/c mice bearing 4T1 tumor. NIR fluorescence images (λ_{ex} = 750 nm, λ_{em} = 780 nm) were dynamically taken under a multifunctional imager (Fusion FX7 Spectra, VILBER, France). Apart from fluorescence imaging, the biodistribution of Au and Pt were further monitored by using inductively coupled plasma-atomic emission spectrometry (ICP-AES). Briefly, major organs and tumor were excised from tumor bearing mice at predefined time points

post-injection. Then, all the samples were lysed by aqua regia, and the amount of Au/Pt was thereby determined based on calibration curves via ICP-AES.

To investigate the pharmacokinetics *in vivo*, fresh blood was withdrawn from retro-orbital venous plexus, followed by homogenization. At last, the element content in blood was similarly quantified by using ICP-AES.

Photoacoustic (PA) imaging capacity

Before exploring the PA response of APS, photothermal conversion ability was first assessed *in vitro*. Briefly, APS NPs (3 mL) at various concentrations in a quartz vial were irradiated by a fibre-coupled semiconductor diode NIR laser (808 nm, 1W/cm^2) for 10 min. A thermal imaging camera (Fluke, TiS55) was utilized to monitor the temperature elevation.

PA images of APS NP dispersion at different concentrations (15.6~600 μg/mL) were scanned by using a Vevo LAZR-X multimodal imaging system (VisualSonics Inc., Canada) *in vitro*. For *in vivo* PA imaging, tumor-bearing mice were intravenously injected with APS NPs (100 μL, 1 mg/mL in saline), and PA images of tumor region was captured at 0, 24 and 48 h post-injection. All the images were processed and quantitatively analyzed by using the same imaging system.

Antitumor effect *in vivo*

Upon the tumor volume reached 250 mm^3, all the mice were allocated into 4 groups (n = 7 each group) at random, and intravenously injected with saline (100 μL), APS NPs (100 μL, 1 mg/mL in saline) or APS NPs (100 μL, 2 mg/mL in saline). Afterwards, tumor volume and body weight were dynamically recorded for 14 days. Tumor growth inhibition (TGI) was indexed according to equation (4).

$$\text{TGI} = (V_c - V_T)/V_c \times 100\% \quad (4)$$

where V_C represents the tumor volume of saline group, and V_T is donated as the tumor volume in treatment groups.

On day 14, all the mice were euthanized to harvest solid tumor and vital organs, which were further processed for histopathological analysis by hematoxylin-eosin (H&E), terminal deoxynucleotidyl transferase-mediated dUTP-biotin nick end labeling (TUNEL), DHE and Ki67 staining.

Statistical analysis

All the results were displayed as mean ± standard (SD), as indicated. One-way analysis of variance (ANOVA) was carried out for the comparisons of multiple groups, while student t test was performed for two-group comparisons. The default thresholds for statistical significance were $^{*}p<0.05$, $^{**}p<0.01$ and $^{***}p<0.005$.

References

1 Wang Q, Chen Q, Yang YH, Shao ZZ. Effect of various dissolution systems on the molecular weight of regenerated silk fibroin, Biomacromolecules 2013; 14: 285-289.

2 Rockwood DN, Preda RC, Yucel T, Wang XQ, Lovett ML, Kaplan DL. Materials fabrication from bombyx mori silk fibroin, Nat. Protoc. 2011; 6: 1612-1631.

3 Mao B, Liu C, Zheng W, Li X, Ge R, Shen H, Guo X, Lian Q, Shen X, Li C. Cyclic cRGDfk peptide and chlorin e6 functionalized silk fibroin nanoparticles for targeted drug delivery and photodynamic therapy, Biomaterials 2018; 161: 306-320.

4 Zhang AM, Huang CS, Shi HY, Guo WW, Zhang X, Xiang HK, Jia T, Miao F, Jia NQ. Electrochemiluminescence immunosensor for sensitive determination of tumor biomarker CEA based on multifunctionalized flower-like Au@BSA nanoparticles, Sens. Actuator, B 2017; 238: 24-31.

5 Murawala P, Phadnis SM, Bhonde RR, Prasad BLV. In situ synthesis of water dispersible bovine serum albumin capped gold and silver nanoparticles and their cytocompatibility studies, Colloid Surf.,B 2009; 73: 224-228.

6 Tang Y, Yang T, Wang Q, Lv X, Song X, Ke H, Guo Z, Huang X, Hu J, Li Z, Yang P, Yang X, Chen H. Albumin-coordinated assembly of clearable platinum nanodots for photo-induced cancer theranostics, Biomaterials 2018; 154: 248-260.

7 Hu JJ, Chen Y, Li ZH, Peng SY, Sun Y, Zhang XZ. Augment of oxidative damage with enhanced photodynamic process and MTH1 inhibition for tumor therapy, Nano Lett. 2019; 19: 5568-5576.

8 Khoubnasabjafari M, Ansarin K, Jouyban A. Reliability of malondialdehyde as a biomarker of oxidative stress in psychological disorders, Bioimpacts 2015; 5: 123-127.

9 Perry SW, Norman JP, Barbieri J, Brown EB, Gelbard HA. Mitochondrial membrane potential probes and the proton gradient: a practical usage guide, Biotechniques 2011; 50: 98-115.

Supplementary figures

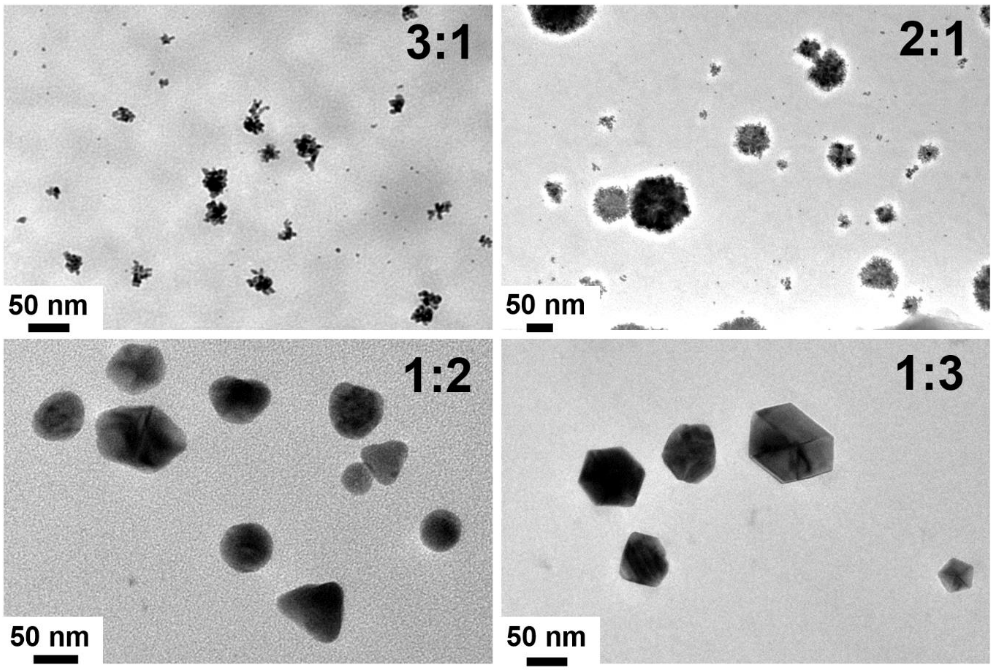

Figure S1. TEM images of APS NPs prepared from the precursors of Au and Pt at different molar ratio (3:1, 2:1, 1:2 and 1:3).

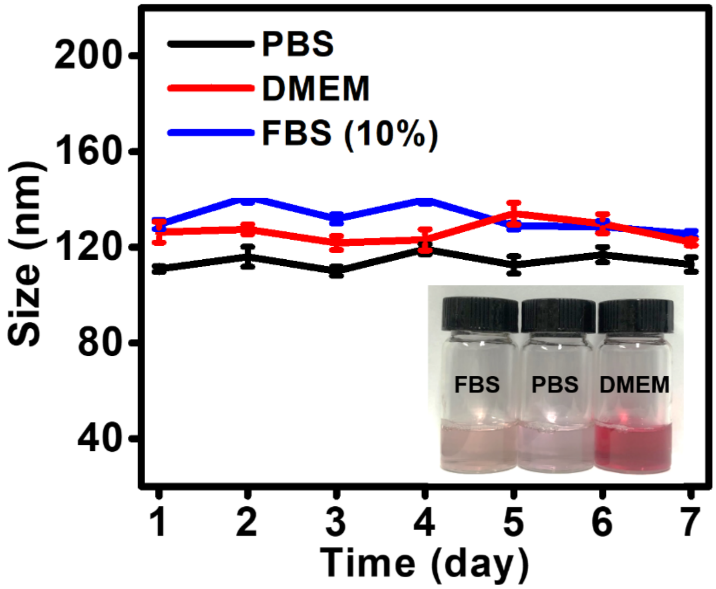

Figure S2. Long-term stability of APS NPs under physiological conditions, evaluated by monitoring the hydrodynamic size in PBS, DMEM and FBS (10%) for seven days.

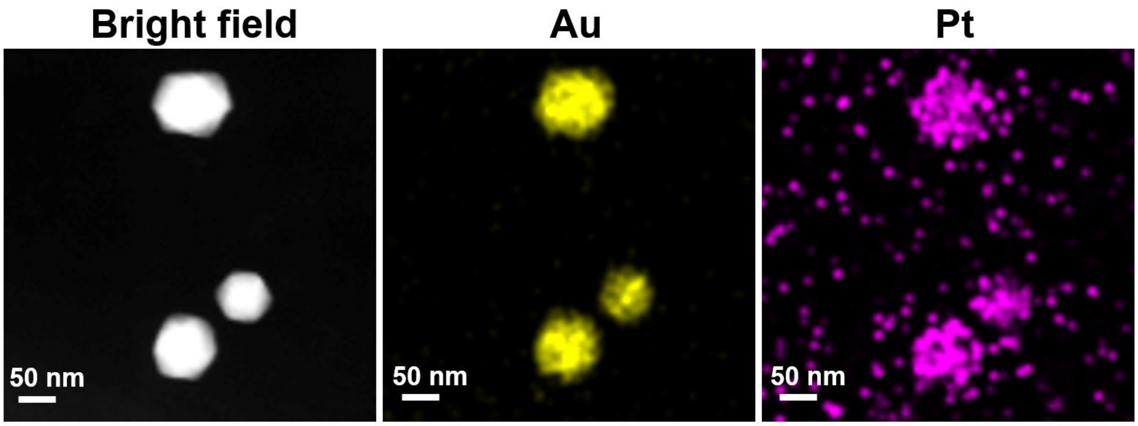

Figure S3. TEM image and EDS element mapping of APS NPs.

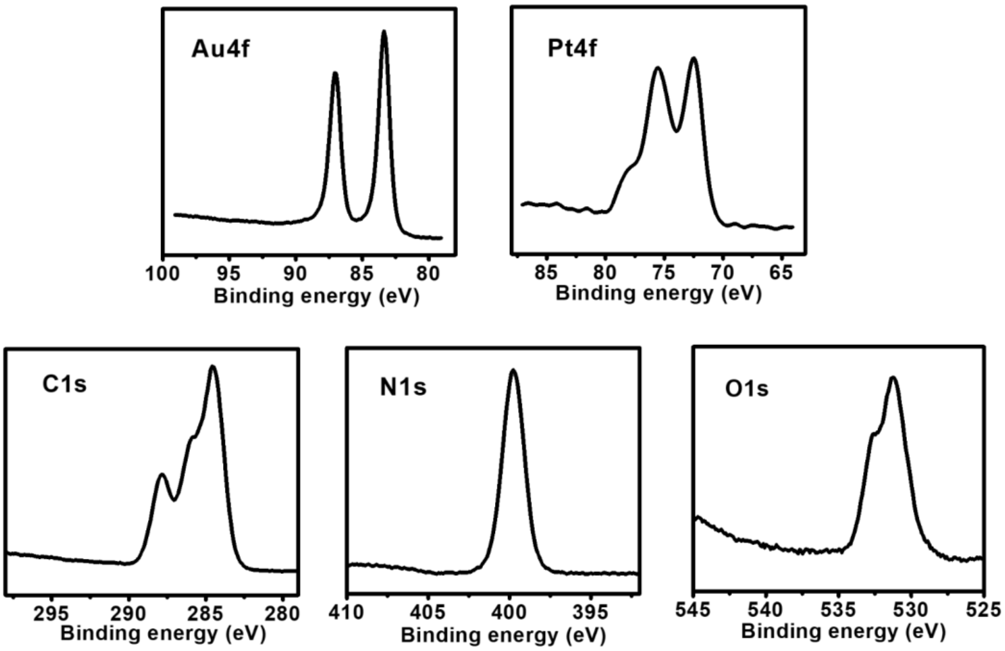

Figure S4. Core level XPS spectra of Au4f, Pt4f, C1s, N1s and O1s in APS NPs.

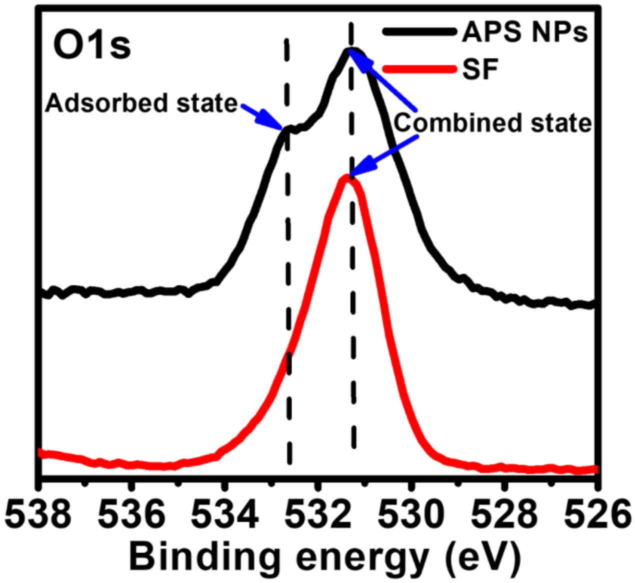

Figure S5. Core level XPS patterns (O1s) of APS NPs and SF.

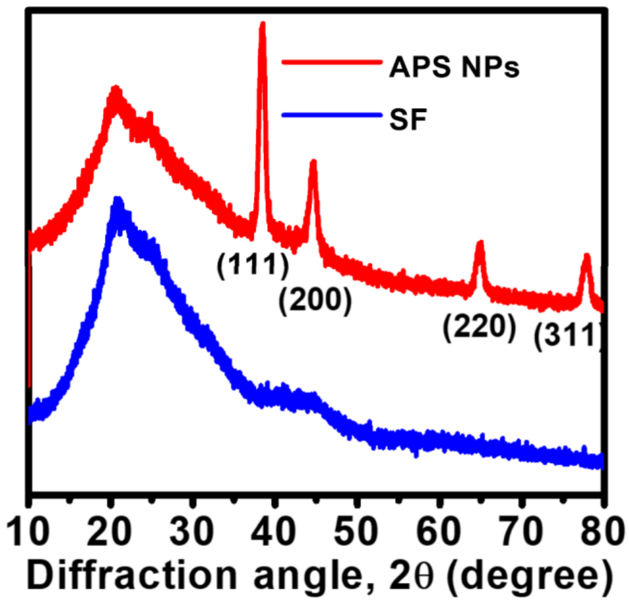

Figure S6. XRD patterns of SF powder and APS NPs.

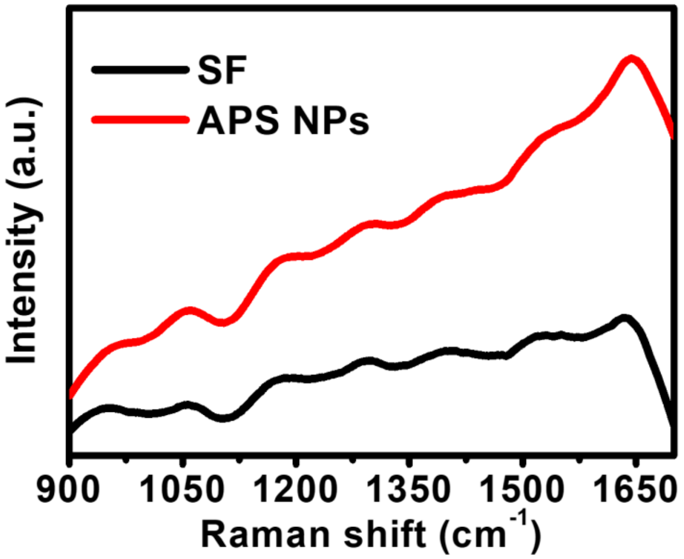

Figure S7. Raman spectra of SF powder and APS NPs.

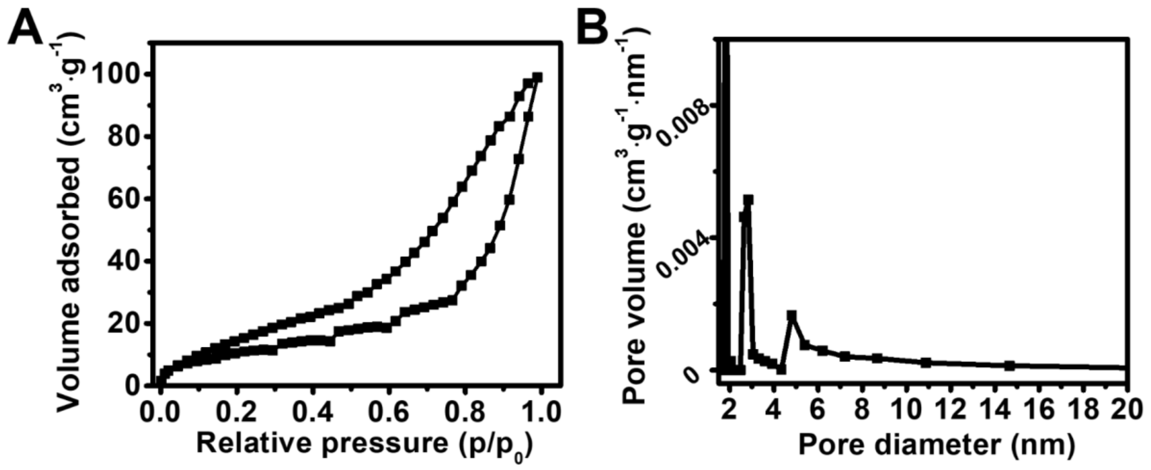

Figure S8. (A) N_2 adsorption–desorption isotherm and (B) the corresponding pore size distribution of the APS NPs;

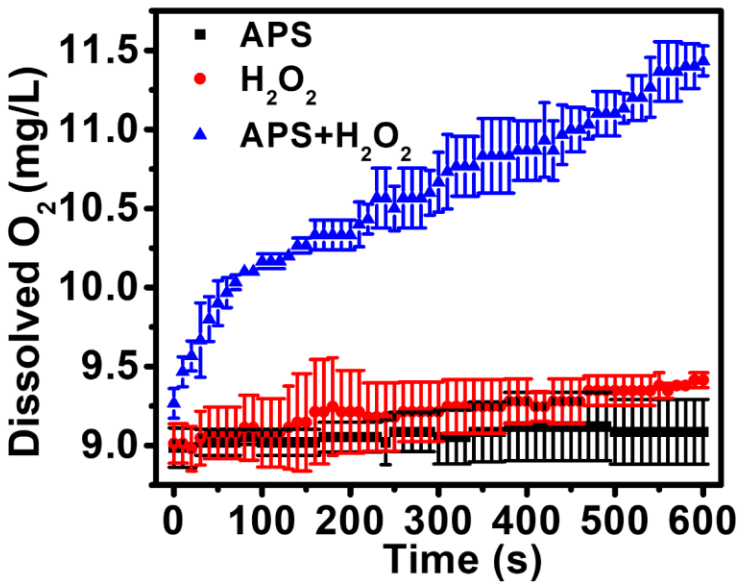

Figure S9. Dissolved oxygen level in PBS (0.1 M, pH = 5.5) containing H_2O_2 (10 mM) and APS (40 μg/mL) during an incubation for 10 min. Compared to control groups, the amount of dissolved O_2 progressively increased with the incubation time, verifying the catalase-mimicking activity of APS NPs.

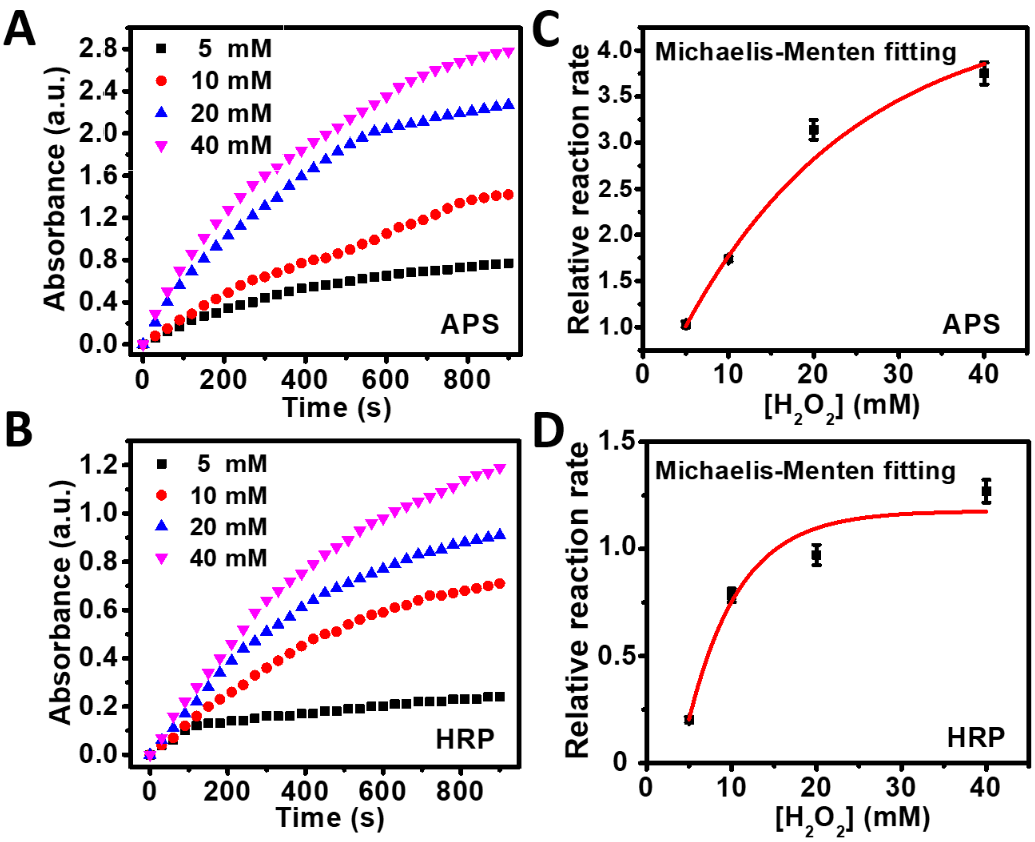

Figure S10. Time course absorbance of (A) APS NPs and (B) HRP (with the equivalent dosage of 40 μg/mL) containing OPD (500 μg/mL) at 435 nm in the presence of H_2O_2 at gradient concentrations; Michaelis-Menten curves for (C) APS NPs and (D) HRP.

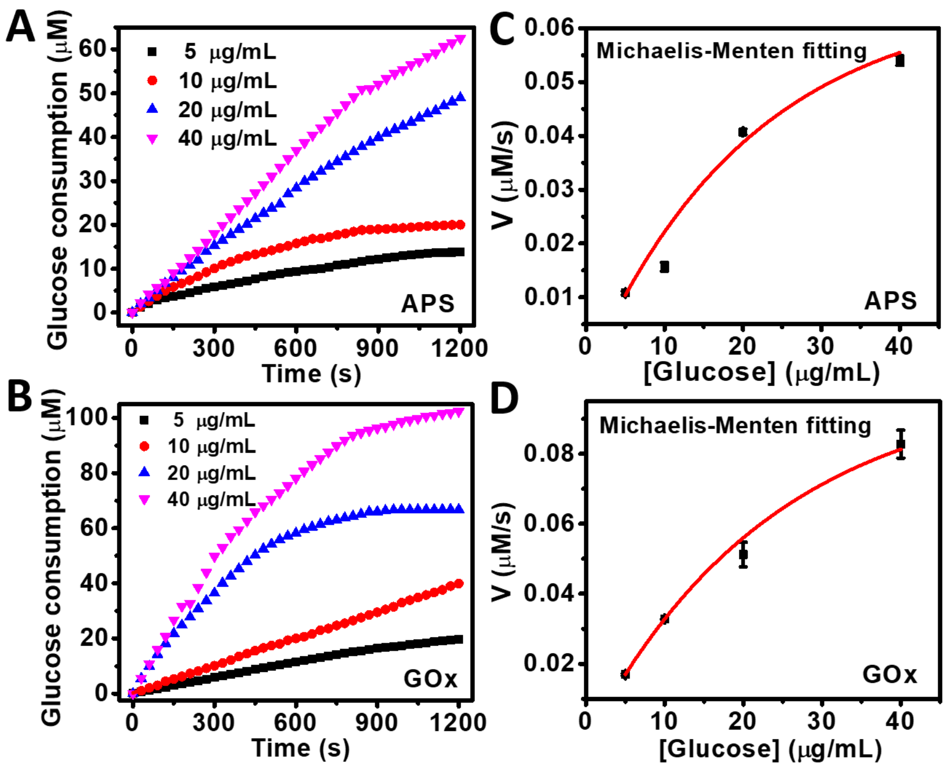

Figure S11. Time-course glucose consumption catalyzed by (A) APS NPs and (B) GOx (with the equivalent dosage of 40 μg/mL) measured using DNS (λ_{max} = 532 nm) on the basis of spectrophotometry in the presence of glucose at gradient concentrations; Michaelis-Menten curves for (C) APS NPs and (D) GOx.

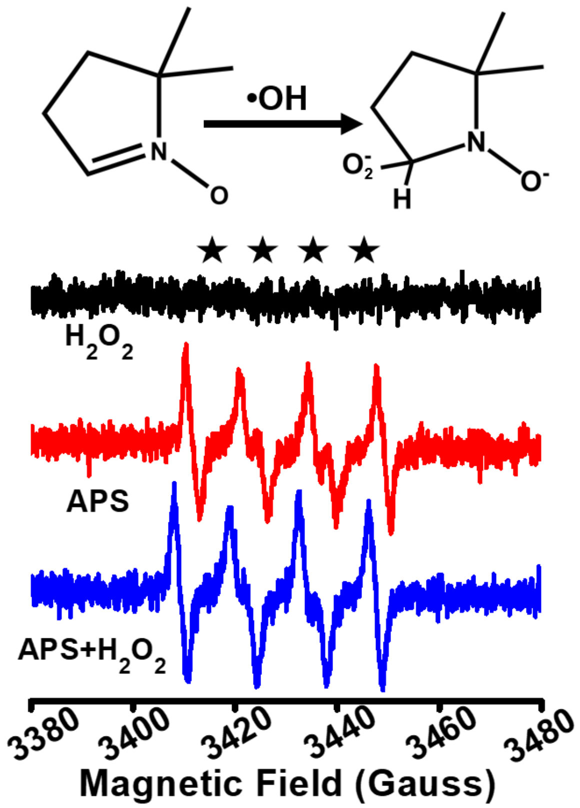

Figure S12. ESR spectra of DMPO/$^{\bullet}O_2^-$ adducts obtained from different samples in methanol.

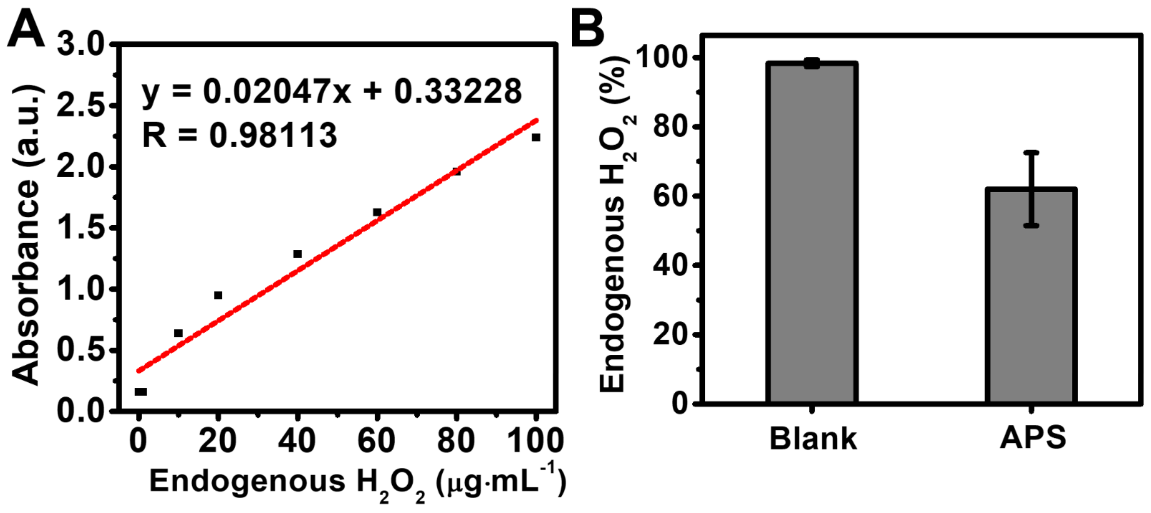

Figure S13. H_2O_2 consumption test. (A) Calibration curve from H_2O_2 quantitative assay kit; (B) level change of H_2O_2 after incubation with APS (40 μg/mL) for 15 min.

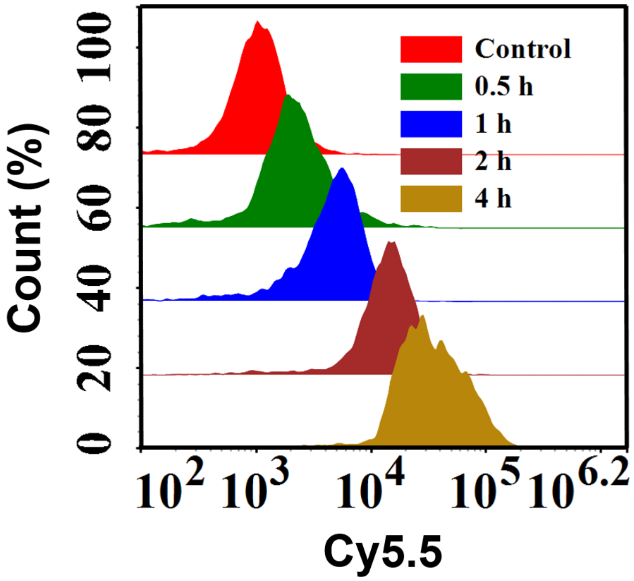

Figure S14. Cellular uptake of APS NPs by 4T1 cells through flow cytometry analysis.

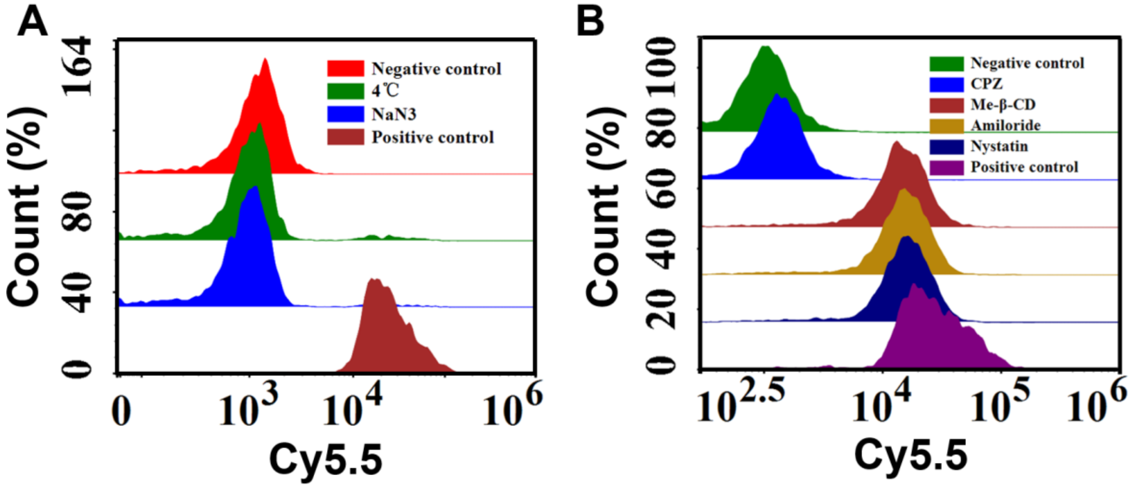

Figure S15. Flow cytometry analysis of APS uptake by 4T1 cells after being treated by (A) energy restriction and (B) receptor-mediated restriction endocytosis factors.

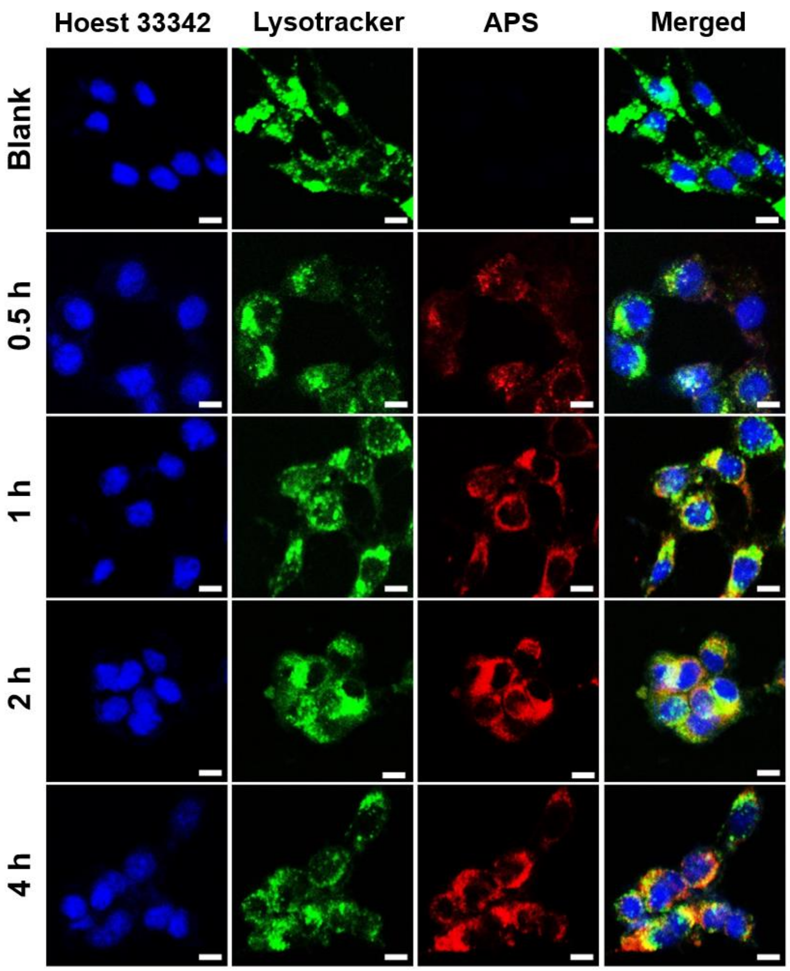

Figure S16. Confocal imaging of 4T1 cells after being treated by Cy5.5-labeled APS NPs (200 μg/mL) for various periods (0.5, 1, 2 and 4 h), and stained by Lysotracker Green (scale bar: 10 μm).

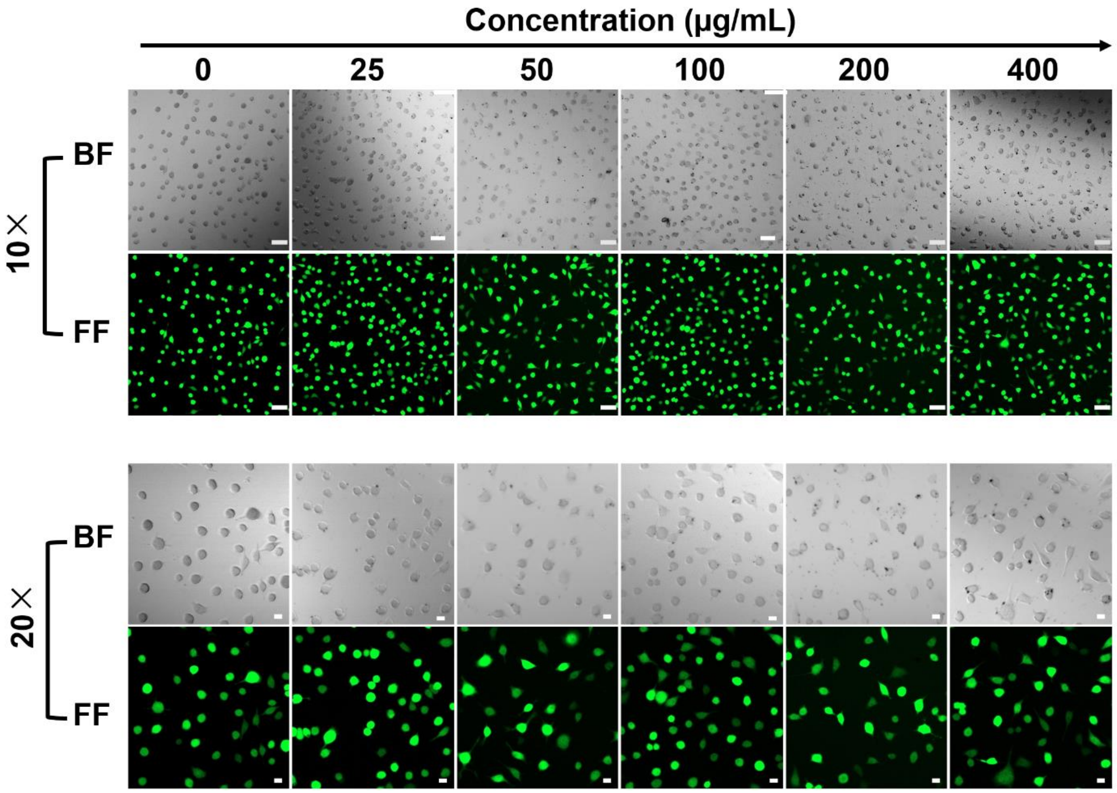

Figure S17. Bright field (BF) and fluorescent field (FF) imaging of L929 cells after being treated by APS NPs at various concentrations for 12 h and processed by LIVE/DEAD fluorescence staining. Scale bar: 50 µm (10×) and 20 µm (20×).

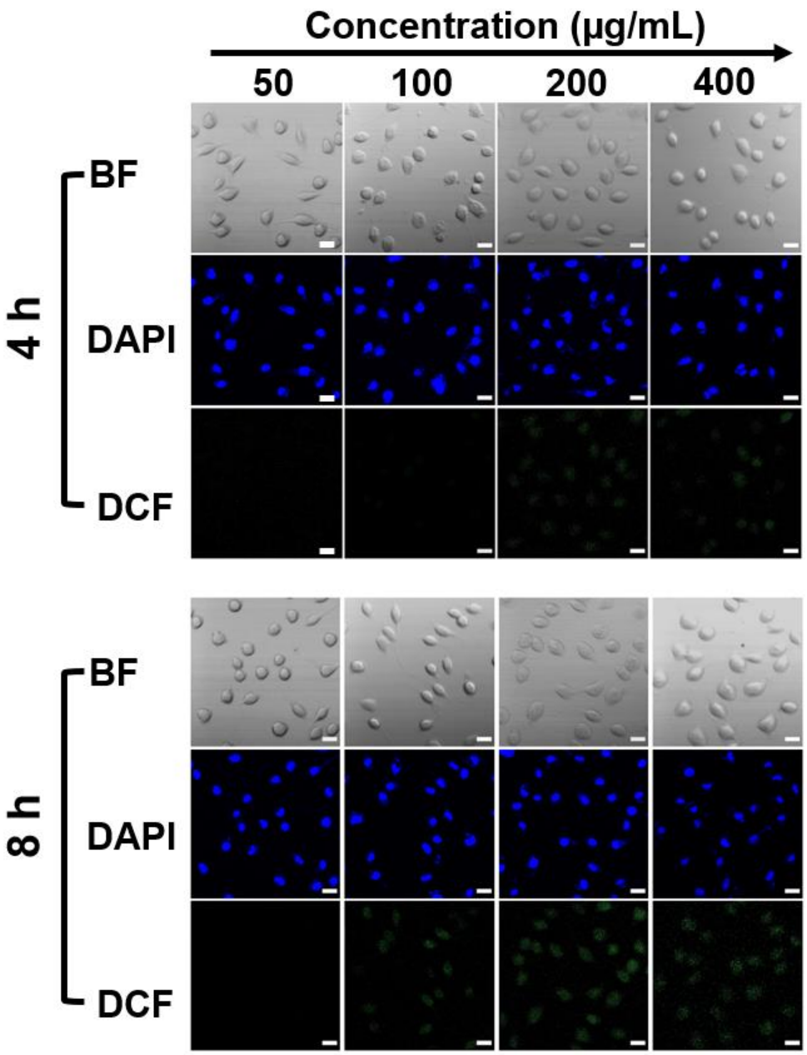

Figure S18. Microscopic images of L929 cells treated by APS NPs and stained by DCFH-DA (scale bar: 20 μm).

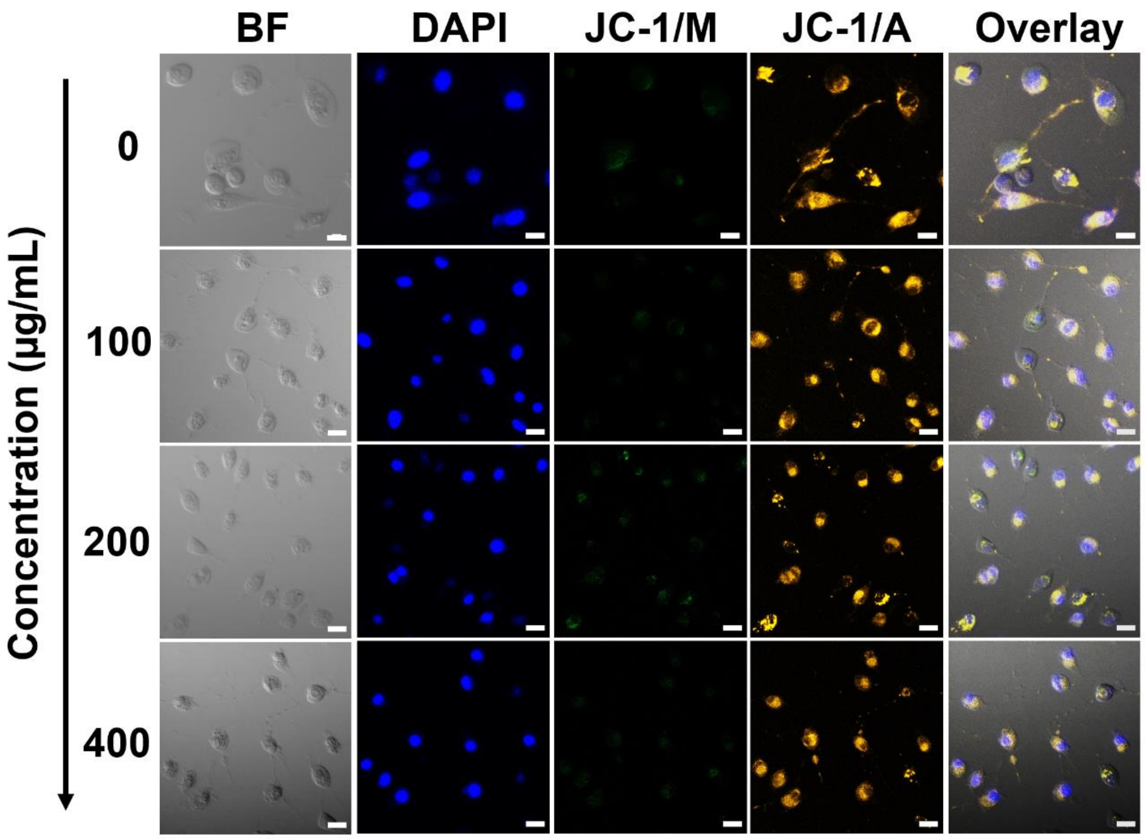

Figure S19. Mitochondrial membrane potential (MMP) of L929 cells after being exposed by APS NPs at various concentrations for 12 h, characterized by JC-1 staining (scale bar: 20 μm). JC-1/M and JC-1/A donated the monomer and aggregation form of JC-1 molecules, respectively.

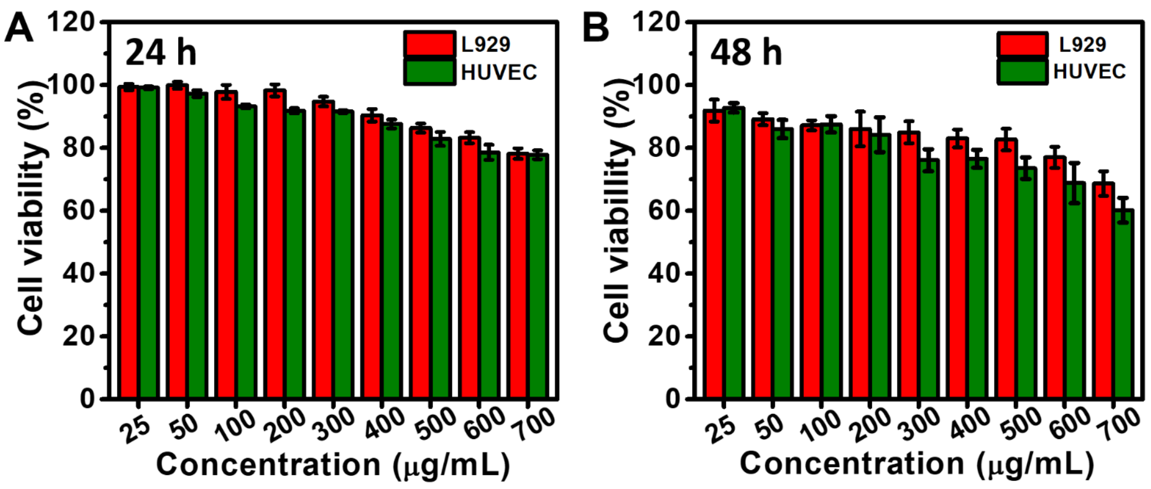

Figure S20. Viability of L929 and HUVEC cells after being treated by APS NPs at different concentrations for (A) 24 h and (B) 48 h.

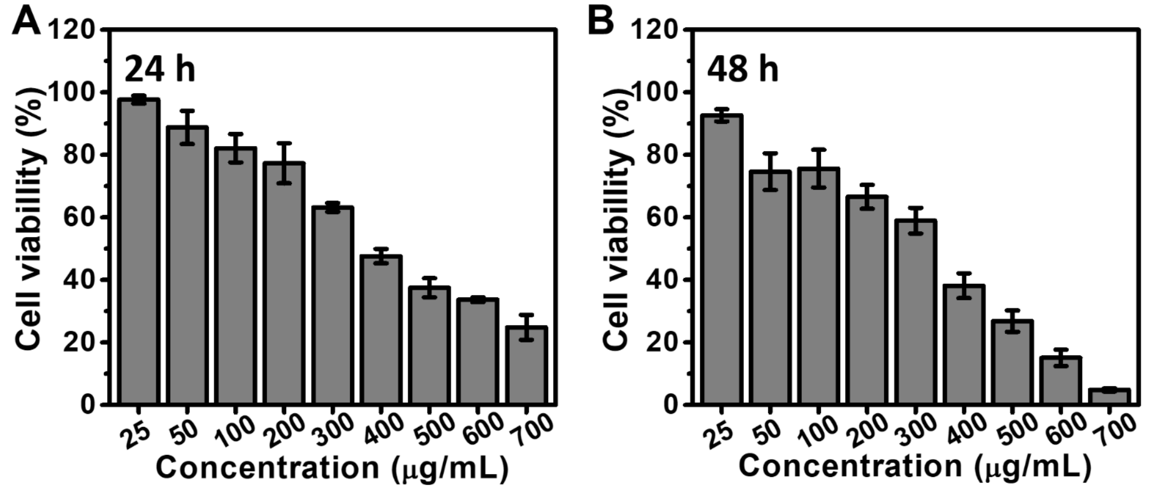

Figure S21. Viability of 4T1 cells after being treated by APS NPs at different concentrations for (A) 24 h and (B) 48 h.

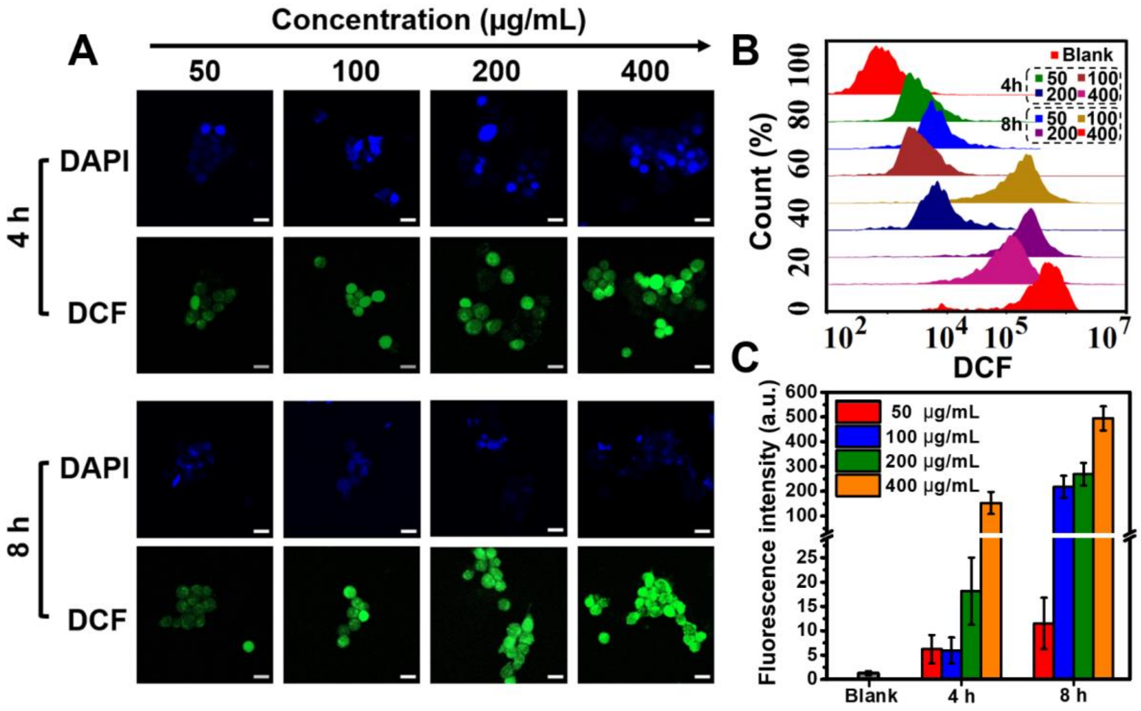

Figure S22. (A) Fluorescence images of 4T1 cells after the treatment of APS NPs and stained by DCFH-DA; (B, C) quantitative fluorescence emission of intracellular DCF through flow cytometry analysis corresponding to (A).

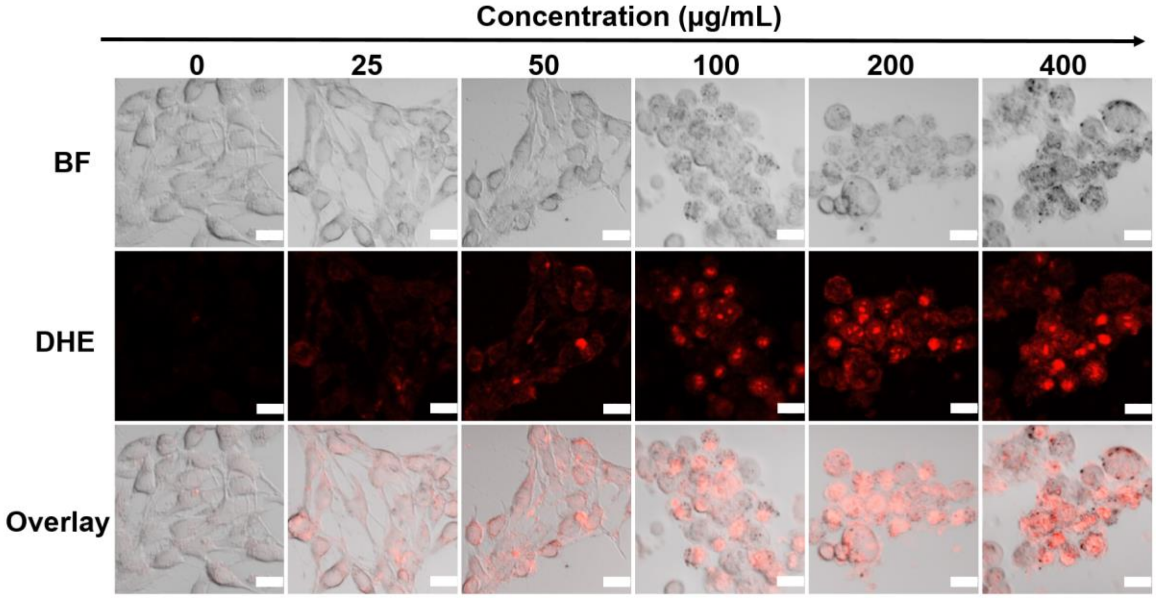

Figure S23. Microscopic images of 4T1 cells treated by APS NPs for 8 h and stained by DHE (scale bar: 10 μm),

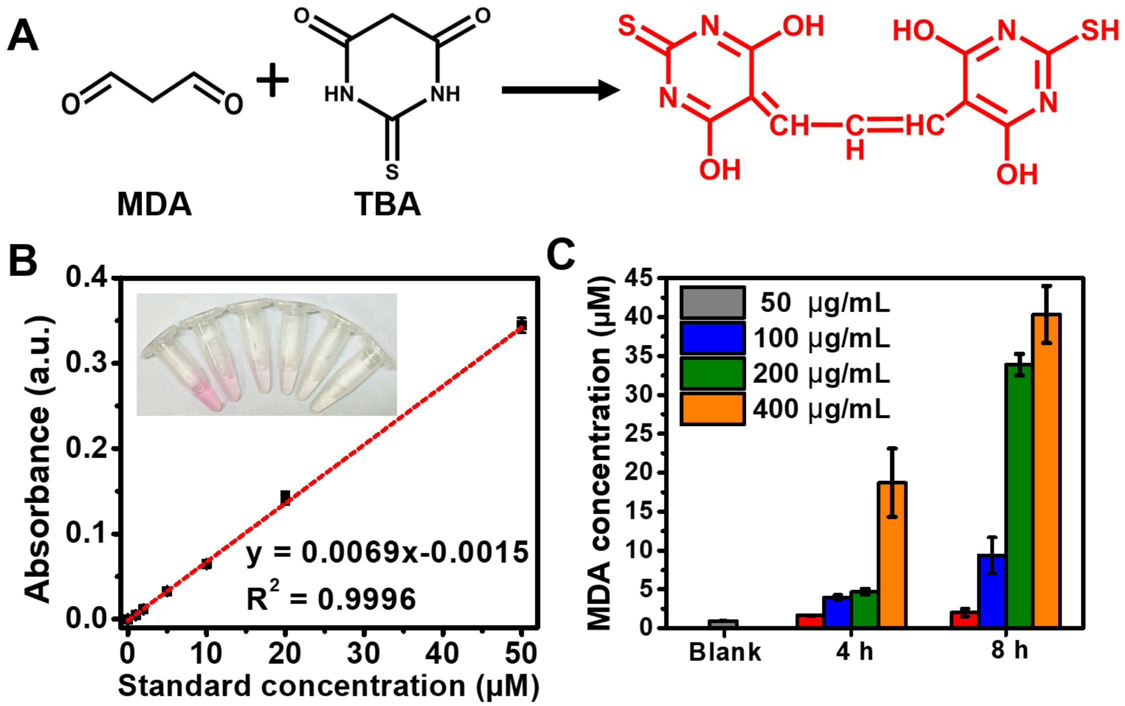

Figure S24. MDA assay. (A) Detection mechanism of cell membrane lipid damage; (B) calibration curve of MDA concentration as the function of optical absorption upon the addition of TBA; (C) intracellular MDA concentration of 4T1 cells after being treated by APS NPs.

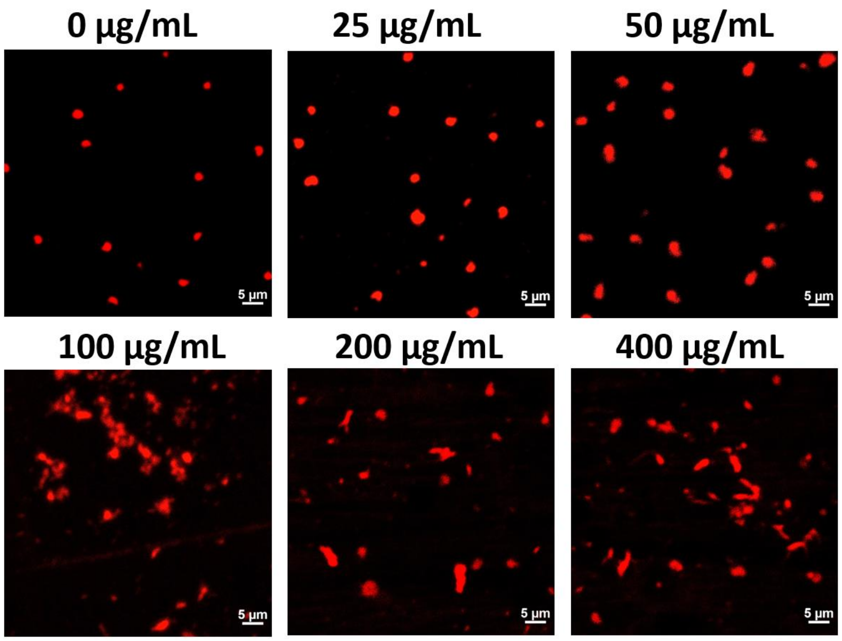

Figure S25. DNA damage assessment of 4T1 cells after being administered by APS NPs for 12 h and processed by Comet assay.

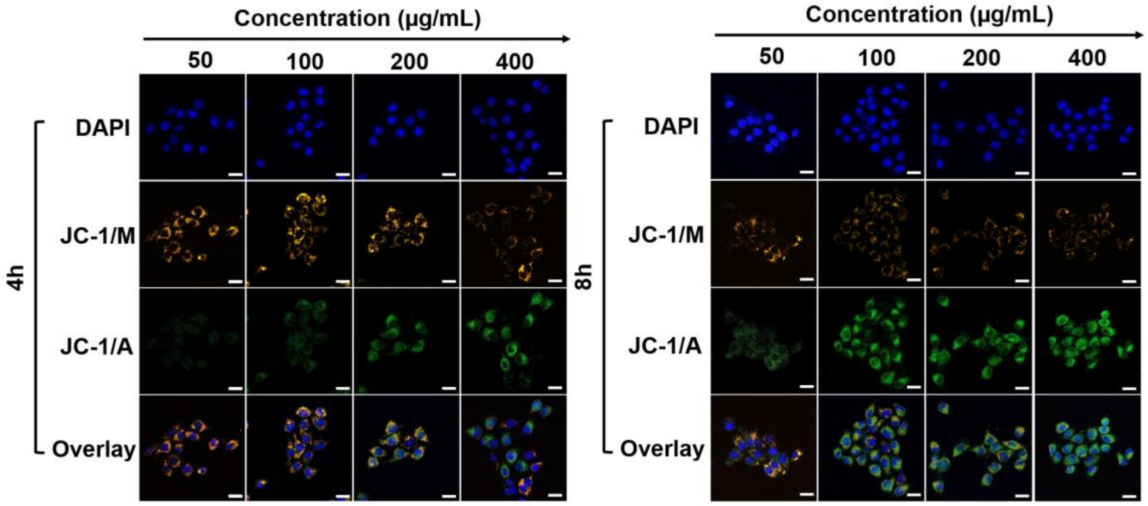

Figure S26. Mitochondrial membrane potential (MMP) of 4T1 cells after being exposed by APS NPs, characterized by JC-1 staining (scale bar: 20 μm). JC-1/M and JC-1/A donated the monomer and aggregation form of JC-1 molecules, respectively.

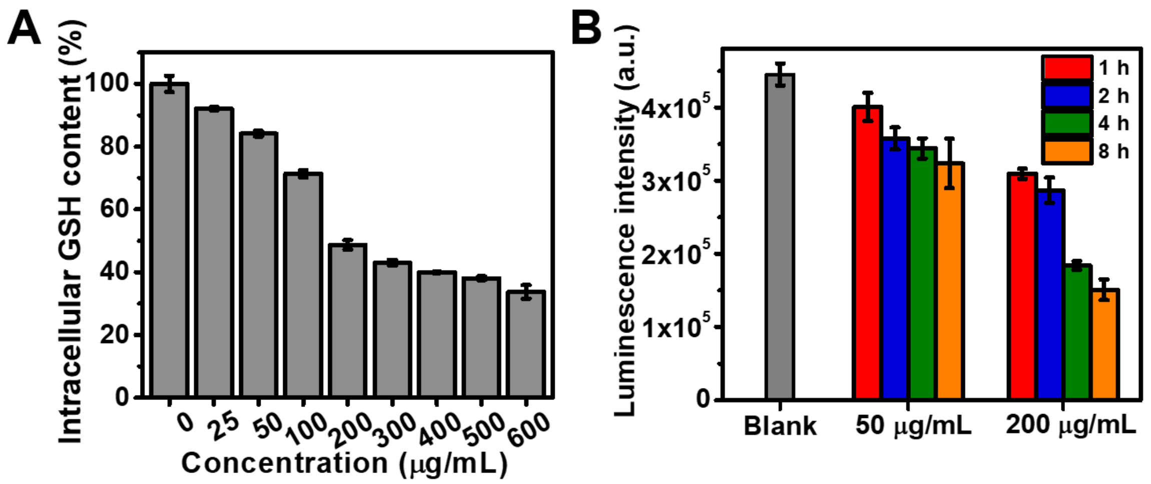

Figure S27. (A) Intracellular GSH content in 4T1 cells after treated by APS NPs for 4 h; (B) ATP content in APS-treated 4T1 cells characterized by ATP assay kit.

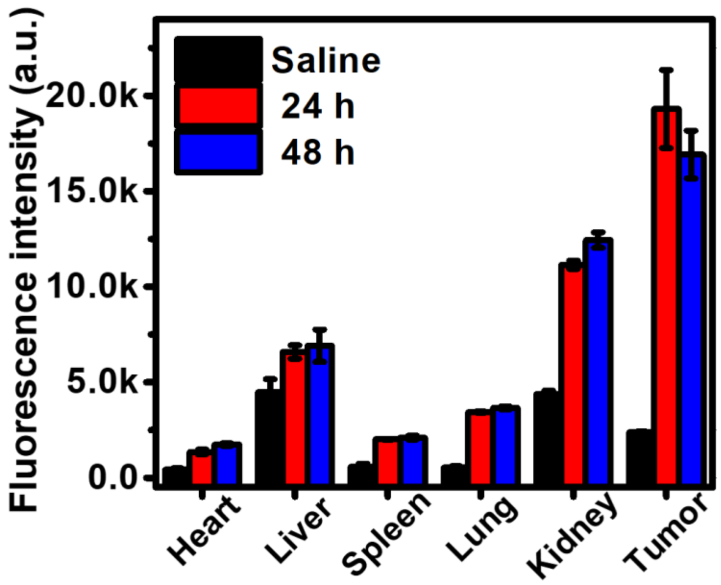

Figure S28. Fluorescence intensity of tumor and vital organs corresponding to Figure 4A.

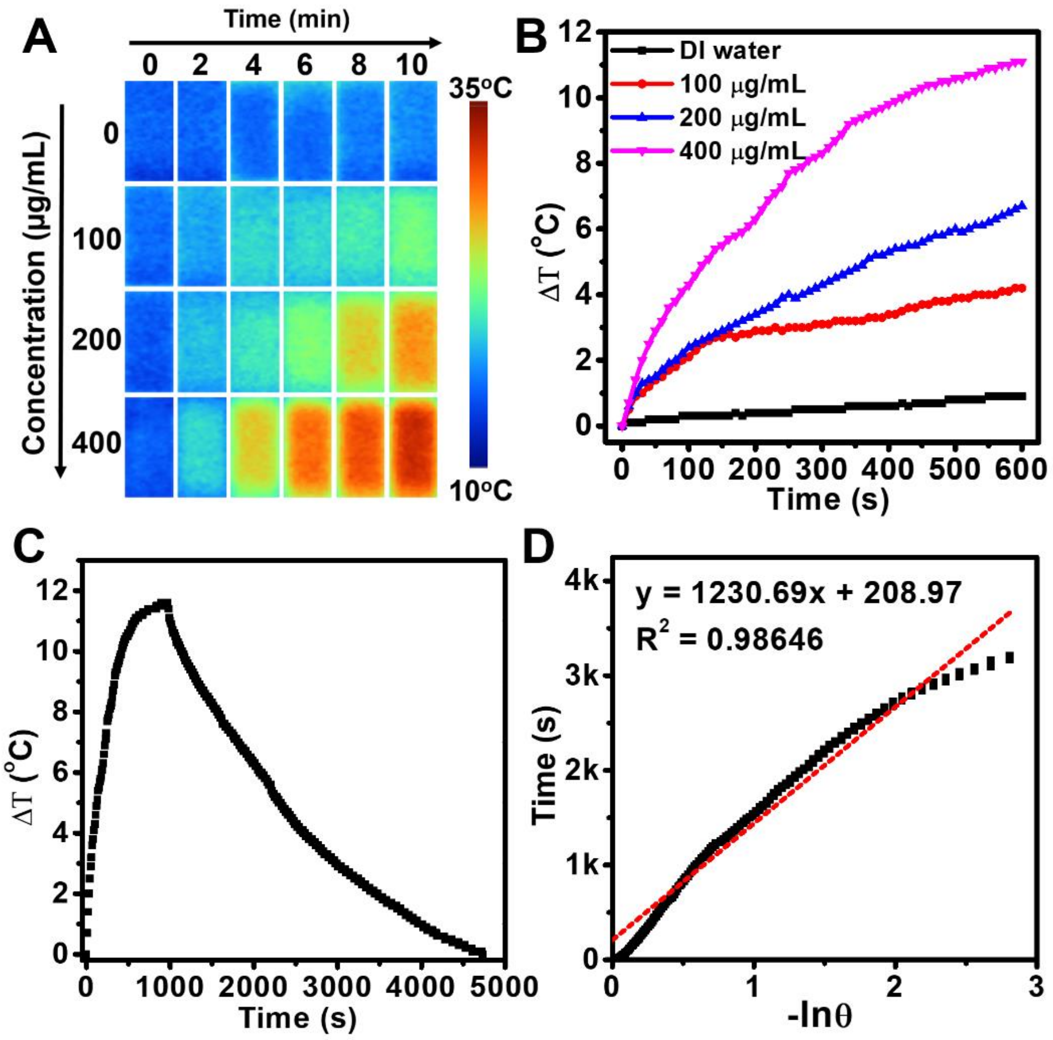

Figure S29. Photothermal property evaluation. (A) Infrared thermographic image of APS NPs upon NIR light irradiation (808 nm, 1W/cm^2); (B) NIR-induced temperature elevation of APS NPs measured by digital thermometer; (C) heating and cooling curve of the dispersion of APS NPs (1 mL, 400 μg/mL); (D) fitting curve of time as a function of -ln(θ) derived from the cooling stage in (C). Photothermal conversion efficiency was calculated to be 9.05%.

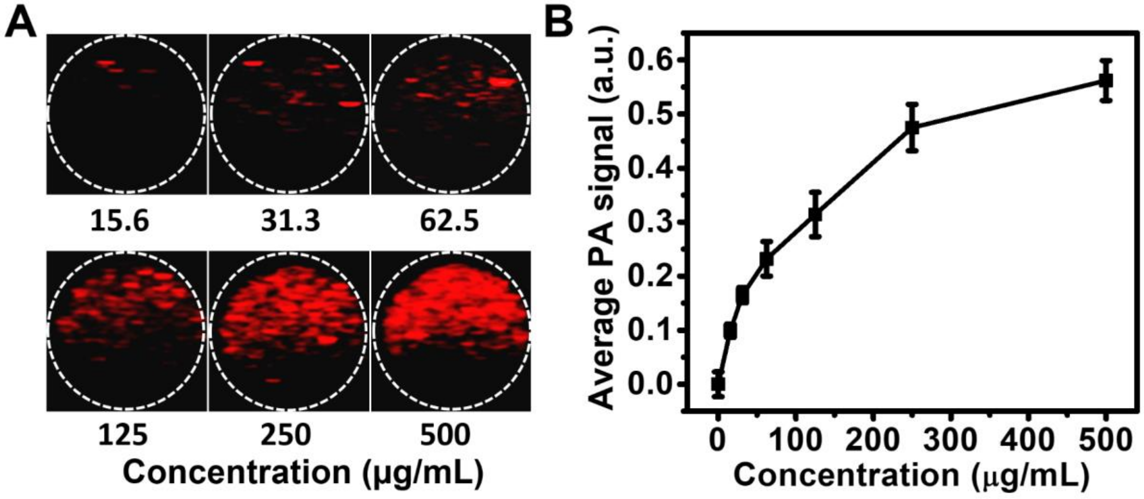

Figure S30. (A) PA images and (B) average PA signal intensity of APS NPs at different concentrations.

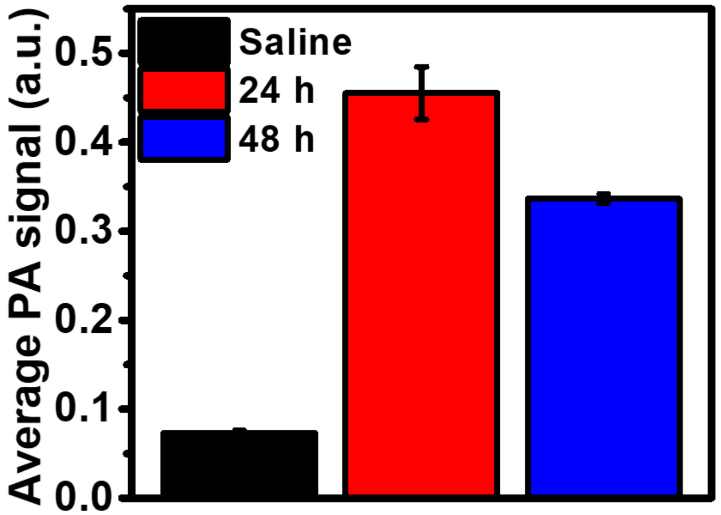

Figure S31. Average PA signal intensity corresponding to Figure 4(B).

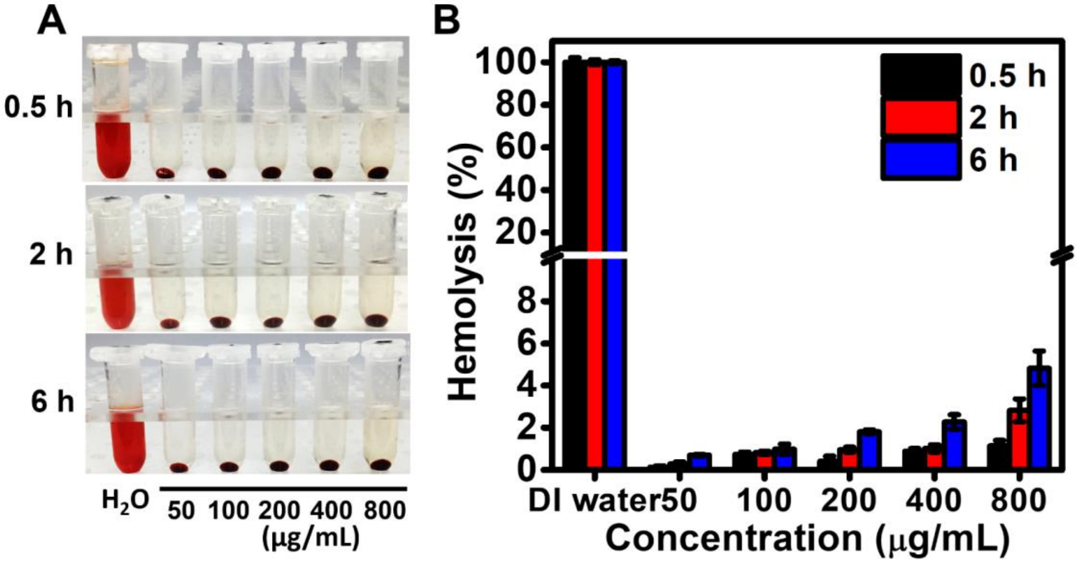

Figure S32. Hemolysis tests by incubating red blood cells (RBCs) with APS NPs at various concentrations. (A) photographs; (B) hemolysis rates.

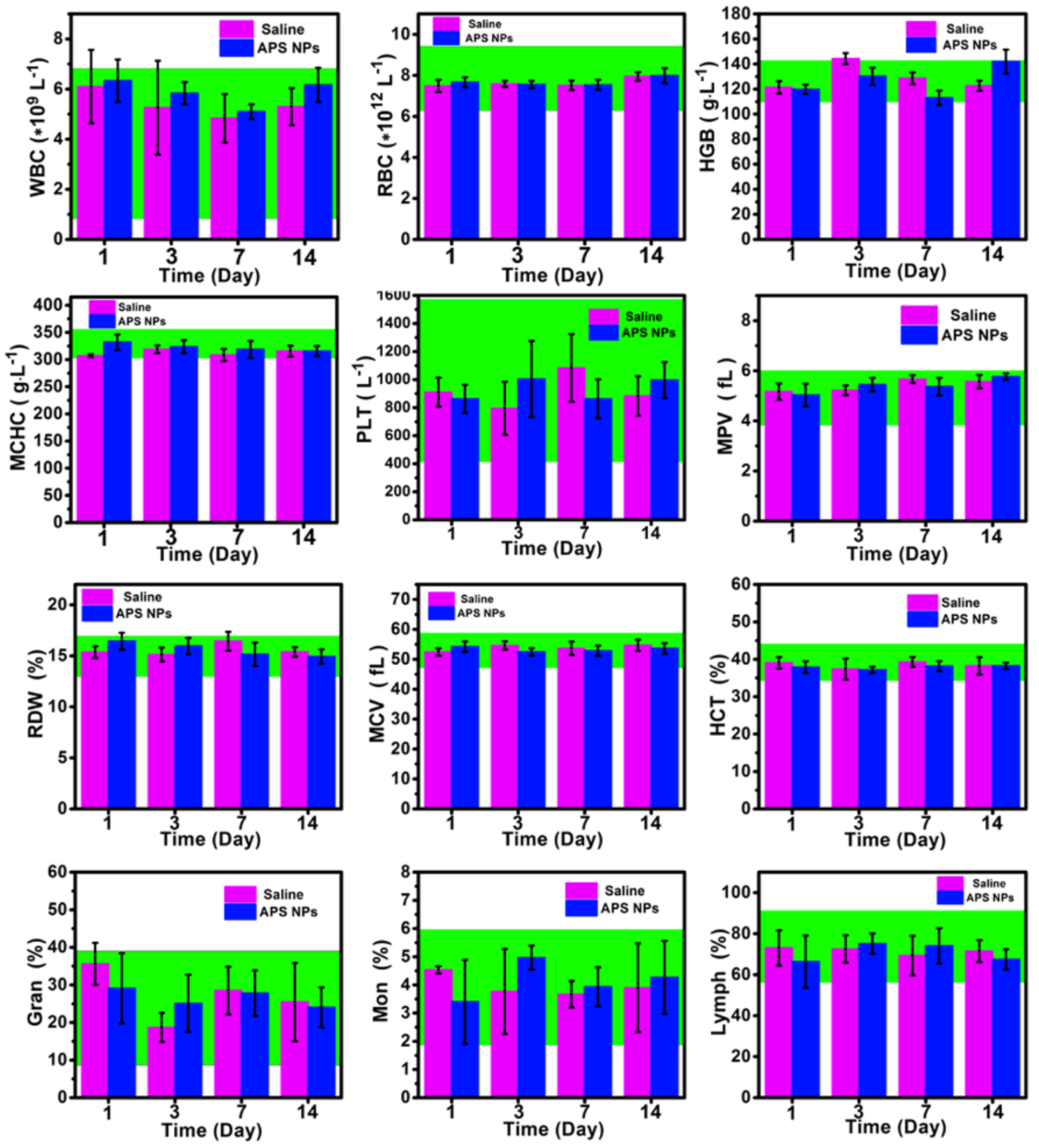

Figure S33. Primary indicators of blood routine test after Kunming (KM) mice intravenously injected with saline or APS NPs (100 μL, 10 mg/mL). The green hatched areas represent the normal reference ranges of hematology data.

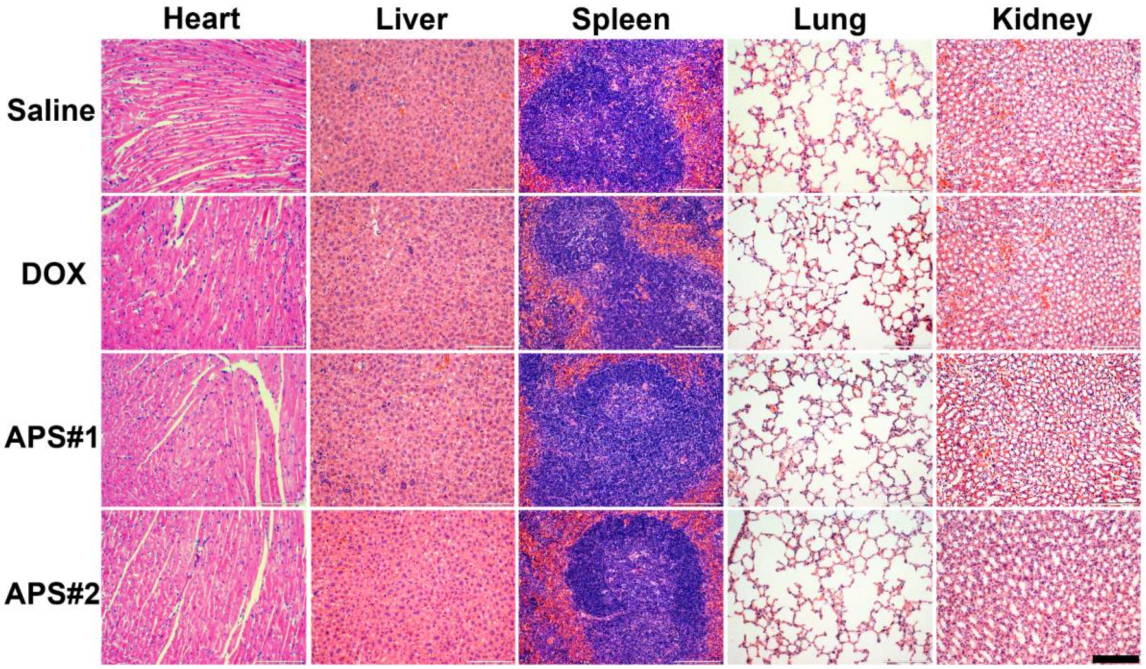

Figure S34. Histological analysis of major organs (heart, liver, spleen, lung and kidney) by H&E staining on day 14 post-injection (scale bar: 200 μm).

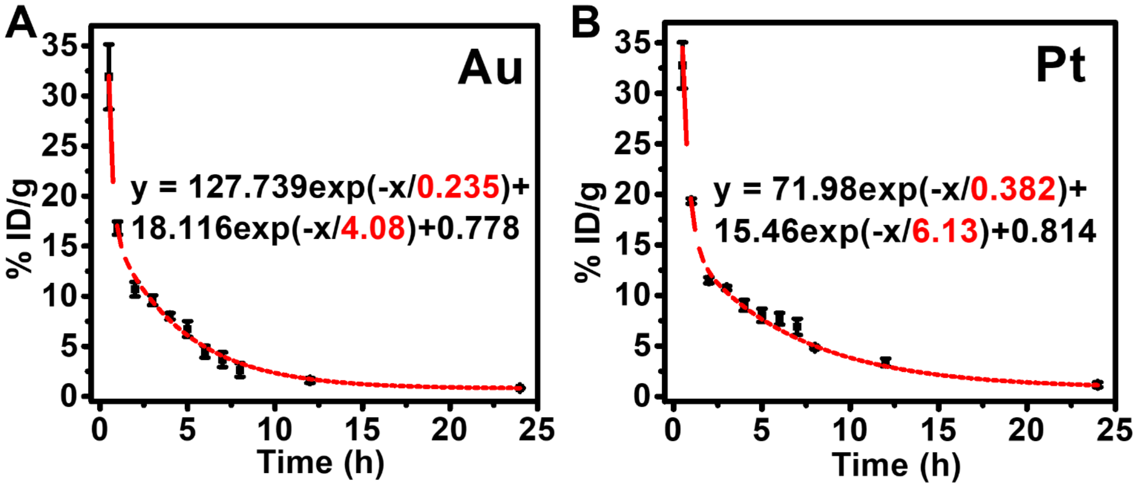

Figure S35. *In vivo* pharmacokinetic curves of (A) Au and (B) Pt over 24 h after intravenous injection of APS NPs in BALB/c mice bearing 4T1 tumors.
